# An integrated platform for 2‐D and 3‐D optical and electrical mapping of arrhythmias in Langendorff‐perfused rabbit hearts

**DOI:** 10.1113/JP287815

**Published:** 2025-06-25

**Authors:** Jimena Siles, Vinícius Silva, Tainan Neves, Italo Sandoval, Angélica Quadros, Giovanni Weber, Óscar Barquero, Ilija Uzelac, João Salinet

**Affiliations:** ^1^ HEartLab, Federal University of ABC São Paulo Brazil; ^2^ Graduate Program in Biotechnoscience Federal University of ABC São Paulo Brazil; ^3^ Graduate Program in Biomedical Engineering Federal University of ABC São Paulo Brazil; ^4^ Virginia Commonwealth University, School of Medicine VA USA; ^5^ Samsung R&D Brazil São Paulo Brazil; ^6^ Rey Juan Carlos University Fuenlabrada Madrid Spain

**Keywords:** accurate diagnosis, cardiac mapping, electrical mapping, electrocardiographic imaging, panoramic optical mapping, torso‐tank setup

## Abstract

**Abstract:**

Electrophysiological mapping is essential for understanding these mechanisms and guiding therapeutic treatments. However, approaches such as invasive electrical mapping, body surface mapping and electrocardiographic imaging face challenges, including low spatial resolution, far‐field interference and signal processing limitations. By contrast, panoramic optical mapping, using fluorescent dyes, offers high spatial resolution and allows direct measurement of cellular action potential *ex situ*. Can the integration of panoramic optical mapping with electrical mapping overcome the limitations of the above‐cited techniques and provide deeper insights into arrhythmic mechanisms? To investigate this, we developed an experimental setup that combines 3‐D panoramic optical mapping with multi‐electrode epicardial electrical mapping and non‐invasive electrical mapping (torso‐tank setup) for electrocardiographic imaging in Langendorff‐perfused rabbit hearts. Our results confirm the feasibility of using simultaneous optical and electrical mapping under sinus rhythm, as well as in atrial and ventricular arrhythmias, using time, frequency and phase analyses. During sinus rhythm and ventricular tachycardia, wavefront propagation showed concordance between modalities, where diverges are observed for atrial arrhythmias. Dominant frequency analysis could recover the frequency of activation better than the inverse of cycle length, and outcomes from all mapping modalities agreed. Reconstructed electrograms presented a good similarity compared to electrograms. By correlating optical and electrical mapping, clinically relevant arrhythmia markers and targets for ablation, from invasive and non‐invasive mapping can be better understood and localised. This platform could also serve as a test bed for studying drug effects, connecting changes from cellular action potential levels to whole‐heart electrophysiology.

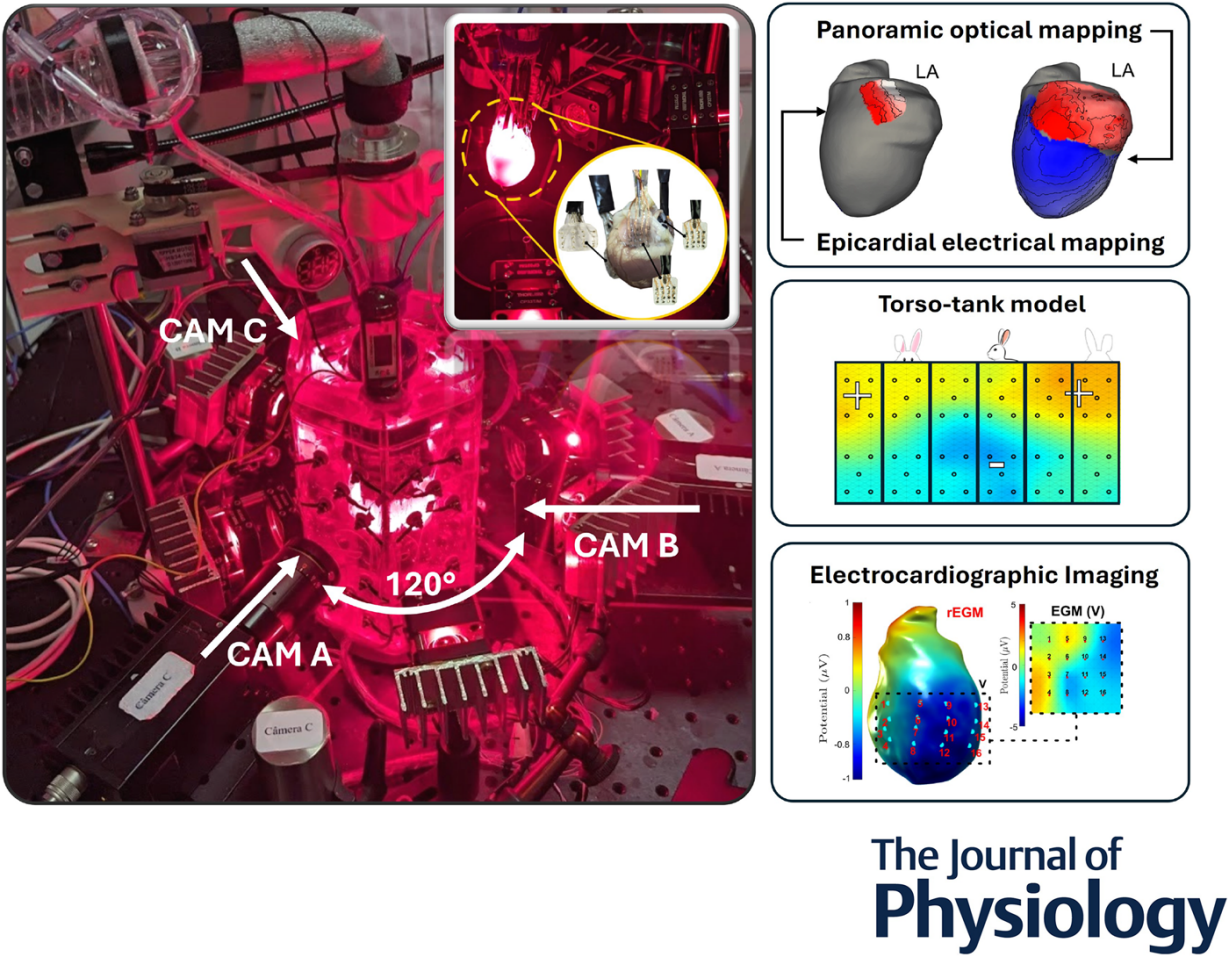

**Key points:**

- Cardiac arrhythmias are still a significant challenge in electrophysiology, with advancements in experimental and clinical research improving our understanding of mechanisms and target for ablation.
- Current electrical mapping technology, both invasive and non‐invasive, is used in science and by commercial systems to identify arrhythmic episodes and mechanisms, but has several limitations mimicking the true electrophysiology behaviour.
- Optical mapping uses fluorescent dyes to measure transmembrane action potentials with high spatial resolution. When combined with electrical mapping, it can enhance cardiac arrhythmia studies and mapping technologies.
- A novel 3‐D platform that integrates panoramic and electrical mapping techniques (epicardium, non‐invasive torso‐tank and electrocardiographic imaging) is presented and validated in isolated rabbit hearts, highlighting that the mapping strategies do not always agree, helping to further improve commercial systems.

## Graphical abstract

**Abstract figure legend** Integrated multimodal platform for panoramic cardiac mapping in isolated heart experiments. On the left, an image of the experimental setup during data acquisition showing a Langendorff‐perfused rabbit heart surrounded by three optical cameras (CAM A, B and C) positioned 120° apart, each coupled with high‐power LEDs for panoramic optical mapping. The setup also includes contact electrical mapping using epicardial multi‐electrode arrays (MEAs, inset top right) and non‐contact electrical mapping via a torso‐tank model (right). On the right, from top to bottom: a typical activation 3‐D map acquired via epicardial electrical (left) and optical (right) mappings; an illustrative torso‐tank potential map; and a comparison between electrocardiographic imaging (ECGi) and contact‐based MEA‐derived voltage maps.

## Introduction

Cardiac arrhythmias are central to clinical electrophysiology, with accurate diagnosis and effective treatment remaining ongoing research priorities (Kumar et al., 2020; McDonagh et al., 2021). Understanding the complex dynamics of cardiac electrical activity is essential for characterising various types of arrhythmias (De Groot et al., 2022). Despite significant advances in the understanding of arrhythmic mechanisms, challenges persist, particularly in relation to different treatment approaches and the low success rates observed in certain patient groups.

Electrical activity during arrhythmic episodes can be mapped using catheter electrodes in either unipolar or bipolar configurations. Although unipolar mapping captures the sum of the currents beneath the electrode (Eckardt & Breithardt, 2009), it is prone to far‐field effects, making bipolar recordings preferable in clinical practice. Bipolar mapping focuses on local activity as deviations from a flat baseline (Eckardt & Breithardt, 2009); however, the morphology of bipolar electrograms (EGMs) depends on the direction of wavefront propagation relative to electrode orientation. This propagation direction can be assessed using EGMs or through three‐dimensional (3‐D) colour‐coded maps based on voltage, activation time or variables obtained by signal processing techniques applied to ECGMs. In addition to invasive mapping, cardiac electrical activity can be measured non‐invasively via a standard 12‐lead electrocardiogram (ECG) or a high‐density electrode placed around the torso, providing improved spatial resolution.

Researchers have successfully recorded heart electrical activity using both invasive and non‐invasive body surface mapping in humans (Guillem et al., 2009; Vanheusden et al., 2019) and animal models (Bear et al., 2018, 2019; Bergquist et al., 2021). This dual approach allows for direct comparison between invasive and non‐invasive mapping. By utilising 3‐D models of both the heart and torso, body surface signals can be estimated through the forward solution (Bergquist et al., 2021), whereas EGMs can be reconstructed using the inverse solution (Bear et al., 2018). These techniques contribute to a better understanding of cardiac electrophysiology.

Another advanced method for recording heart activity is optical mapping, often regarded as the gold standard. This technique uses fluorescent dyes that attach to cardiac cell membranes, acting as probes to measure transmembrane action potentials (APs) directly and with high spatial resolution. Alternatively, calcium probes can measure intracellular calcium responses to membrane depolarisation. High‐speed cameras capture these APs or calcium transients with high spatial resolution without the need for direct contact with cardiac tissue (Holcomb et al., 2009; Laughner et al., 2012; Lou et al., 2011). Optical mapping offers several advantages over electrical electrode‐based mapping and has become a valuable validation tool (Bear et al., 2019; Berenfeld et al., 2011; Stoks et al., 2023).

Unlike EGMs obtained through electrode contact, which measure extracellular potentials, the fluorescence signal from optical mapping is directly proportional to the transmembrane potential. Moreover, optical mapping is not sensitive to wavefront propagation direction (a difficulty in bipolar mapping), has reduced influence on far‐field effects compared to unipolar mapping and offers significantly higher spatial resolution than both bipolar and unipolar electrical mapping. A notable advancement in optical mapping is panoramic optical mapping, which uses multiple high‐speed cameras around the heart to capture electrical activity from various viewpoints (Gloschat et al., 2018; Lee et al., 2017). This approach generates detailed fluorescence images of electrophysiological activity across the entire epicardial surface, enabling researchers to study the spatio‐temporal propagation of electrical signals responsible for maintaining arrhythmias. The development of an integrated platform comprising electrical mapping (invasive and non‐invasive) and panoramic optical mapping would be of great importance to improve the current knowledge of cardiac arrhythmias and contributing to improve the current commercial system used to guide catheter ablation. In the present study, we present an experimental setup that combines simultaneously 3‐D panoramic optical mapping with multi‐electrode epicardial electrical mapping and non‐invasive electrical mapping (torso‐tank setup) for ECG imaging (ECGi) in Langendorff‐perfused rabbit hearts. The combination of these multimapping modalities represents a significant technological advance in experimental cardiac electrophysiology by enabling a comprehensive view of cardiac electrical activity across multiple spatial scales and perspectives. This unique configuration allows for the direct comparison of cellular APs with surface EGMs and reconstructed ECGi signals, enabling mechanistic insights into the initiation and maintenance of arrhythmias, which is not achievable with existing techniques alone.

## Methods

### Local ethical approval

This study was approved by the Ethics Committee on the Use of Animals of the Federal University of ABC in São Paulo, Brazil, under protocol 3947230519. All procedures were carried out according to institutional guidelines for animal experimentation. The rabbits, sourced from an authorised supplier (Granja RG, Suzano, Brazil), were kept in a light/dark cycle and were provided with balanced food supplied by the vendor. Access to fresh and clean drinking water was not restricted. In addition, the system featured air exhaust and continuous climate control (22°C) throughout the pre‐surgical period.

### Isolated perfusion heart

The rabbits were initially lightly anaesthetised with an i.m. injection of buprorenorphine (0.05 mg kg^−1^), followed by an i.m. injection of the Ketamine/Xylazine cocktail after 30 min (50 and 7 mg kg^−1^, respectively) to induce deep anaesthesia. If a rabbit responded to stimuli, such as toe pinching, an additional injection was administered. An anticoagulant, heparin (3500 IU), was i.v. infused into the marginal vein of the rabbit's ear. Ten minutes after the heparin infusion, the hearts were excised via sternal thoracotomy, which involved opening the pericardium and cutting at the upper end of the ascending aorta to remove the heart (Lou et al., 2011). Once extracted, the heart was submerged in a glass plate containing a modified Krebs–Henseleit solution with reduced calcium concentration (in mm): 115 NaCl, 4.6 KCl, 25 NaHCO_3_, 0.5 CaCl_2_.2H_2_O, 1.2 KH_2_PO_4_, 1.2 MgSO_4_.7H_2_O, 1 Na‐pyruvate and 11 glucose C_6_H_12_O_6_). Residual tissues such as the lung, trachea, fat and connective tissues were removed, and any remaining blood was expelled through retrograde aortic perfusion. The heart was then perfused in Langendorff setup through the aorta, maintaining a pressure of 80 mmHg and a flow rate of 20–40 mL min^−1^. The perfusate used was a modified Krebs–Henseleit buffer (Bailey & Ong, 1978) with the following composition (in mm): 115 NaCl, 4.6  KCl, 25  NaHCO_3_, 1.2  CaCl_2_.2H2O, 1.2  KH_2_PO_4_, 1.2  MgSO_4_.7H_2_O, 1  Na‐pyruvate and 11 glucose, bubbled with carbogen (5% CO_2_, 95% O_2_) and adjusted to a pH of 7.4 ± 0.05 (conductivity of 13 ± 1 mS cm^−1^).

### Electrophysiology study

In total, 18 New Zealand White rabbits, with an average weight of 3.4 ± 0.4 kg, were used in the study. For each heart, two balloons were inserted into the atria and inflated to prevent atrial collapse (Ravelli & Allessie, 1997). The first balloon was inserted through the inferior vena cava into the right atrium (RA), and the second through one of the pulmonary veins into the left atrium (LA). When required, for atrial studies, the atrioventricular (AV) node was ablated using an electrically isolated needle, except at the tip, to isolate ventricular electrical activity from atrial activity. The needle was connected to a radio frequency (RF) generator (Stockert 70; Biosense Webster, Norderstedt, Germany), and the AV node was ablated with 15 W of RF power delivered for 10 s. The return electrode (ground) was connected to the aorta. Successful AV block was confirmed by real‐time monitoring of both the atrial and ventricular activity. Optical mapping was conducted using the voltage‐sensitive dye Di‐4‐ANBDQPQ (Matiukas et al., 2007). A stock solution (1 mg mL^−1^ in ethanol) was prepared, and 0.25 mg of the dye was added to 150 mL of perfusate, which was recirculated three or four times until the heart tissue fully absorbed the dye. After dye loading, the perfusion solution was replaced with a dye‐free solution. A small volume (75 mL) of Krebs–Henseleit solution containing a higher concentration of blebbistatin (20 µm) was infused to suppress mechanical contractions and avoiding distortions in the optical mapping images acquisition. For the remainder of the experiment, a lower concentration of 1.7 µm of (–)‐blebbistatin was used to maintain the suppression of contractions (Fedorov et al., 2007). To induce cardiac arrhythmias, a custom‐made bipolar pacing catheter with two electrodes on its tip and 2 mm intra‐electrode spacing was used. The system included a custom‐built, battery‐powered, programmable current‐source stimulator (Aleksa Tech, Petaling Jaya, Malaysia) designed to deliver timed and controlled cardiac stimulation. In the S1–S2 protocol, a train of ten S1 (pulse width of 2 ms) pulses with a 300 ms period was followed by an S2 pulse, with the interval decreasing from 150 to 20 ms until the refractory period was reached (Ravelli & Allessie, 1997). After determining the refractory period, the perfusion solution was switched to a Krebs–Henseleit solution containing carbachol (1 µm) for continuous perfusion. Atrial fibrillation (AF) was induced through burst pacing (S1 protocol), applying a triple train of 100 pulses at 20 Hz, separated by intervals of 1.3 s (Zafalon et al., 2004).

### Setup description

**Figure 1:**
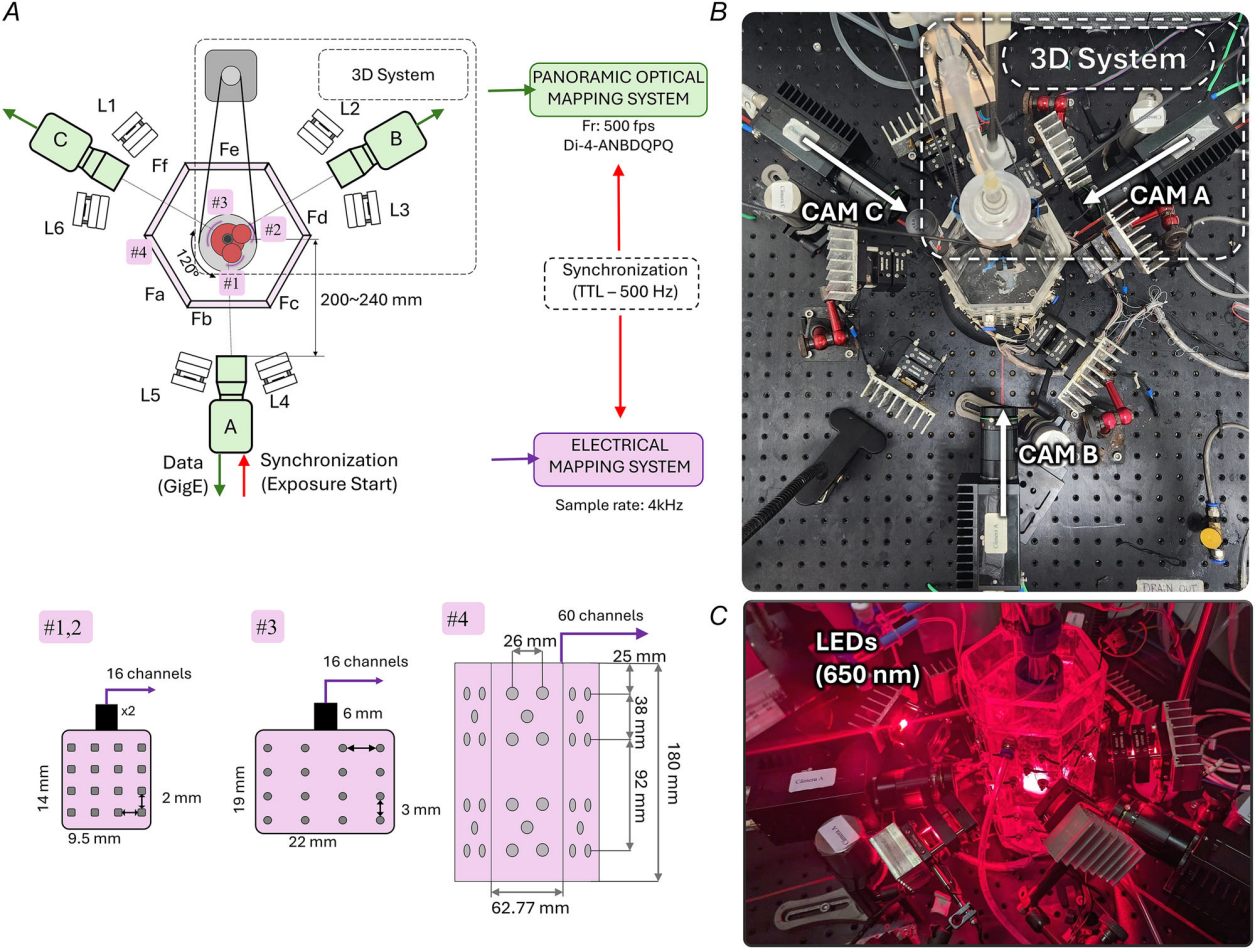
Schematic diagram and photographs of the integrated experimental setup for simultaneous panoramic optical and electrical mapping *A*, schematic representation of the three‐dimensional (3‐D) system integrating the optical mapping system (light green) and the electrical mapping system (light purple). Three high‐speed cameras (CAM A, B and C) are positioned around the Langendorff‐perfused heart for panoramic optical mapping. The electrical mapping system includes three custom‐designed multi‐electrode arrays (MEAs), labelled #1 to #3; and a torso‐tank model for non‐invasive mapping labelled #4. These MEAs are placed over the RA, LA and ventricular surface, respectively. Detailed representations of the MEAs, including their real dimensions, are shown at the bottom of the schematic. *B*, top‐view photograph of the experimental setup mounted on the optical table, highlighting the positioning of the cameras. A dashed box indicates the custom‐built 3‐D support structure designed to acquire the sequence of images for the 3‐D reconstruction with the optical camera A. *C*, photograph of the system during an optical mapping experiment, showing red LEDs (650 nm) illuminating the heart.

This study describes an experimental platform for mapping the cardiac electrical activity in Langendorff‐perfused New Zealand White rabbit hearts. The system combines synchronised high‐speed optical mapping with simultaneous electrical mapping to characterise cardiac arrhythmias (Fig. 1*A*). Electrical mapping includes a torso‐tank model for non‐invasive mapping, with electrodes placed on the tank containing the Langendorff‐perfused heart (torso‐tank rabbit model) and epicardial electrical mapping using customised multi‐electrode arrays (MEAs) in contact with both atria and ventricles. The following section provides a more detailed description of the implemented setup. Figure 1*B* shows the top‐view photograph of the experimental setup mounted on the optical table, highlighting the positioning of the cameras.

#### Epicardial electrical mapping

Three custom‐designed MEAs with 16 electrodes were developed, and placed in the LA (#1), RA (#2) and ventricle surface (#3) for epicardial electrical mapping (Fig. 1*A*, light purple #1, 2 and 3). For the atria, the 16 platinum‐iridium electrodes sourced from a commercial catheter were fixed onto a 4 × 4 configuration and fixed in a polyethylene terephthalate (PET) square of 14 × 9.5 mm with an inter‐electrode distance of 2 mm. The ventricular MEA used the same number of electrodes, but they were arranged on a larger PET square, measuring 19 × 22 mm with an inter‐electrode distance of 3 mm vertically and 6 mm horizontally. Each MEA was grounded via a metal ring fixed between the Langendorff cannula and the heart's aorta. The MEAs were connected to a 64‐channel recording head‐stage (RHD 64 Channel Recording Head‐stages, C3315; Intan Technologies, Los Angeles, CA, USA) via 36‐pin wire adapters (C3420; Intan Technologies). These connections linked MEAs to the Open Ephys acquisition board (https://open‐ephys.org), enabling the acquisition of the epicardial EGMs at a sampling rate of 4 kHz.

#### Torso‐tank model for non‐invasive electrical mapping

The torso‐tank model is designed to simulate the physical properties of the rabbit's torso. This calculation resulted in an estimated volume of ∼1500 cm for the rabbit's torso and the designed tank. The model is a hexagonal tank (side length = 62.77 mm, height = 180 mm) designed for high‐density, non‐invasive electrical mapping (Fuller et al., 2000). Ten unipolar surface‐mounted electrodes were strategically placed on each face of the hexagonal tank (Fig. 1*A*, #4). These electrodes, made of 10 mm diameter stainless steel bolts, were positioned to create clear‐view windows, ensuring unobstructed views of the heart for panoramic optical mapping, as described in the next section. The reference electrode for each surface‐mounted electrode was connected via a soldered metal strip attached to the Langendorff cannula in the aorta. In total, 60 electrodes were connected to a 64‐channel recording head stage (RHD 64 Channel Recording Head stages, C3315; Intan Technologies) through 36‐pin wire adapters (C3420; Intan Technologies). This configuration enabled high‐density electrocardiogram acquisition at a 4 kHz sampling rate, using an Open Ephys acquisition board and the open‐source electrophysiology software. The heart was immersed in the tank and filled with a high‐concentration sucrose solution (23 mm NaCl, 260 mm sucrose, pH 6.8 ± 0.4 and conductivity of 2.23 µS cm^−1^). This solution mimics the conductivity of the animal's chest cavity and was continuously recirculated using a temperature‐controlled (37−38°C) water bath.

#### Panoramic optical mapping

Six deep red (650 nm) LEDs (Luminus Devices, Sunnyvale, CA, USA) provided uniform illumination of the curved heart surface (Fig. 1*C*). The LED light was collimated using a condenser lens (ACL2520U‐A; Thorlabs, Newton, NJ, USA) and passed through a band‐pass filter (650/40 nm; Thorlabs). Fluorescence emitted from the heart was filtered using a 715 nm long‐pass filter (FF01‐715/LP‐22‐D; Semrock, Rochester, NY, USA) before being captured by three high‐speed cameras (HB‐1800‐S; Emergent Technologies, Austin, TX, USA) equipped with C‐mount 25 mm lenses (CF25ZA; Fujinon, Saitama City, Japan). The intrinsic parameters also include a pixel size of 9 × 9 µm and a sensor size of 1.1´ (17.6 mm CMOS). The lens has a focal length of 25 mm and a distortion smaller than 1% within the region of interest (ROI), which was considered negligible. Cameras were placed at a height of 9 cm above the base, positioned 120° apart around the tank. Their radial distance (*R*) from the tank centre varies between 20 and 24 cm, according to the heart's size, forming an annular arrangement instead of a fixed circular configuration. The co‐ordinates for each camera in the world reference frame are expressed as:

(1) $$Camera\;A:\;\left(R,9,0\right),\mathrm{with}\;20\leq R\;\leq 24\;\mathrm{cm}$$

(2) $$Camera\;B:\;\left(-\frac{R_{2}}{2},9,\frac{\sqrt{3}R_{2}}{2}\right),\mathrm{with}\;20\leq R\;\leq 24\;\mathrm{cm}$$

(3) $$Camera\;C:\;\left(-\frac{R_{2}}{2},9,-\frac{\sqrt{3}R_{2}}{2}\right),\mathrm{with}\;20\leq R\;\leq 24\;\mathrm{cm}$$

All three cameras visualised the ventricles, with additional coverage of the LA by camera A and the RA by camera B. Recordings were acquired simultaneously at 500 frames s^−1^, with a resolution of 1600 × 1000 pixels, 12‐bit depth and a total duration of 10 s (yielding 5000 frames), using StreamPix 9 (NorPix, Montreal, QC, Canada). The cameras were triggered via hardware using the GPI5 input and automatically stopped after 10 s (Fig. 1*A*, light green).

#### 3‐D heart geometry

**Figure 2:**
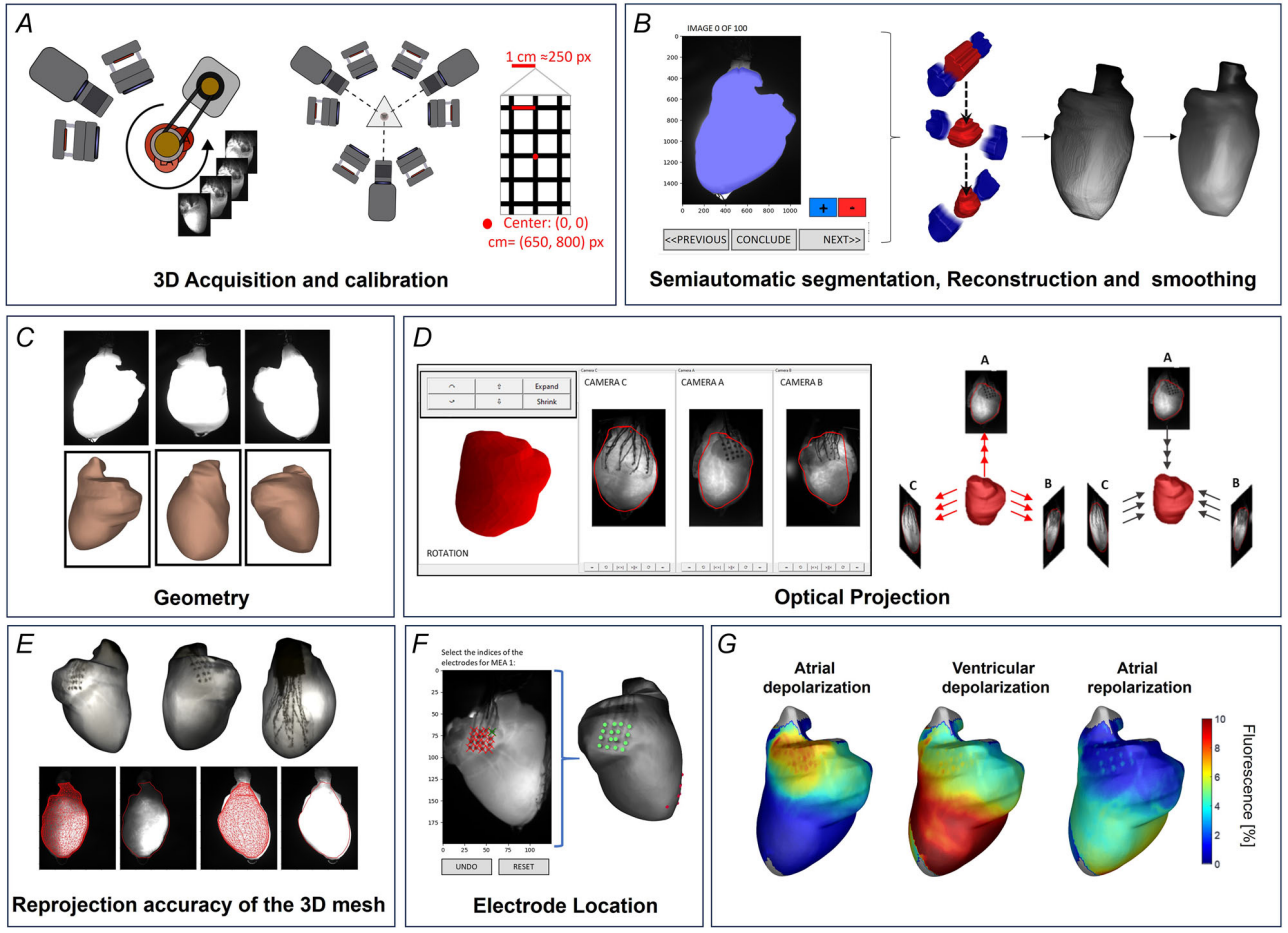
3‐D acquisition and calibration *A*, the optical system acquires images by rotating a single camera 360° around the heart (left). Calibration is performed using a triangular prism placed at the centre of the setup. *B*, segmentation, reconstruction and smoothing: a semi‐automatic segmentation interface (left) identifies the heart silhouette across all frames. The reconstruction process (centre) carves regions of interest (ROIs) to generate the 3‐D mesh. Comparison between initial and smoothed meshes (right). *C*, geometry: refined meshes obtained from the volume boundaries, after applying simplification and filters. *D*, interface (left) used to align and refine the mapping between optical and geometric data. Inverse projection examples from three cameras are shown (right). *E*, reprojection accuracy of the 3‐D mesh: validation of the alignment between 2‐D and 3‐D data using projection contours (bottom row). *F*, electrode location: python interface to manually identify electrodes location using the 2‐D optical data (left) and the already computed electrodes over the 3‐D heart surface (right). *G*, 3‐D surface projections showing atrial depolarisation (left), ventricular depolarisation (centre) and atrial depolarisation (right).

After acquiring the heart's electrophysiology data under cardiac arrhythmias with simultaneous non‐invasive, epicardial contact and optical mapping, the 3‐D heart geometry was reconstructed as follows. One optical mapping camera recorded a sequence of heart images while rotating the heart by 360° in 3.6° increments. Two red LEDs provided uniform illumination for the heart's silhouette segmentation and subsequent 3‐D mesh reconstruction (Fig. 1*A*, dotted square, and Fig. 2*A*, left). The custom setup was 3‐D printed and controlled by an Arduino Uno (https://www.arduino.cc) connected to a bipolar stepper motor driver (DRV8825; Texas Instruments, Dallas, TX, USA) and a stepper motor (JK35HS34‐1004). The motor is connected with the gear, holding the heart through a belt with 3:1 ratio. A custom LabVIEW GUI (National Instruments, Austin, TX, USA) allowed the user to control the rotation in both clockwise and counterclockwise directions. The toolbox driver software Vision Acquisition Software IMAQdx, version 21 (National Instruments) was used to acquire, display and save the images. Calibration was performed using a triangular prism with 15 known landmarks on each face, placed at the heart's position in the centre of the tank. The prism, with grids on its outer surfaces, was oriented toward each camera (Fig. 2*A*, right) (Gloschat et al., 2018). Extrinsic parameters of the camera were determined based on optical prism calibration and predefined values. The focal length was initially set to infinity, with plans for future computation. The optical centre was assumed to be the central pixel of the image. The extrinsic parameters, including the rotation matrix (*R*) and translation vector (*t*), were determined and incorporated into a similarity transformation matrix (*M*), comprising a transformation that combines rotation, dilatation and shift from origin. This matrix maps pixel co‐ordinates to real‐world space, with the origin set at the centre of the tank, ensuring proper alignment for image acquisition. The transformation from pixel space to real‐world co‐ordinates follows the equation:

(4) $$P_{w}=MP_{C}$$

where *Pw* denotes the real‐world co‐ordinates of a point and *Pc* the corresponding pixel co‐ordinates, the transformation is defined by matrix *M*. This matrix is obtained by solving a linear system based on known prism landmarks and their pixel locations. Initially, an affine transformation, *M*, is regularised by removing shear components, resulting in a similarity transformation. Camera orientations are defined by inclination angles (α, β and γ) relative to the tank co‐ordinate system. Since the cameras face the heart without vertical tilt, α = 0°. The angle β is determined by camera positioning: 0° for camera A, 120° for camera B and 240° for camera C. The roll angle is assumed to be γ = 0°.

Following the image acquisition sequence, heart silhouette identification is performed using custom‐developed interactive software in Python, version 3 (https://www.python.org) (Fig. 2*B*, left), each silhouette is our ROI. Then, ROIs are used to carve a knowledge volume (solid cube) along its respective direction (between 0 and 360°), generating an approximated geometry volume of the heart (Fig. 2*B*, middle). Subsequently, voxels in the volume shell are determined by verifying their neighbourhood, and it the respective triangular mesh of the volume is obtained using the Poisson method (Kazhdan et al., 2006). Figure 2*B* (right) illustrates the improvement in mesh quality achieved through surface refinement (five iterations) of a 3‐D reconstructed heart model, displaying the raw mesh obtained from initial segmentation and the same model after applying a smoothing algorithm, resulting in a more anatomically realistic and visually coherent representation.

Figure 2*C* shows three refined meshes, corresponding to each camera, obtained from the volume boundaries, after applying simplification and filters. Assuming a reconstruction with 20,000 vertices covering an average area of ∼2902 ± 400 mm^2^, the average resolution per vertex is around 0.15 ± 0.2 mm^2^, which corresponds to a linear spacing of ∼0.54 ± 0.07 mm between vertices. Figure 2*D* illustrates the alignment process of the final 3‐D heart geometry with the 2‐D optical images obtained from the three cameras during the experiment. Specifically, the 3‐D heart mesh generated in the previous reconstruction steps is projected onto each 2‐D camera view based on the known spatial orientation of each camera relative to the heart. Fine alignment is achieved through iterative rotation and translation of the 3‐D mesh to optimise its correspondence with the recorded images. The projection of the 3‐D geometry onto the 2‐D camera images follows a systematic approach:

- Only the vertices of mesh triangles with a positive normal component in the direction of the respective camera are selected, ensuring that only surfaces facing the camera are visualised. The silhouette (highlighted in red in Fig. 2*D*) is defined by triangles whose normal are nearly perpendicular to the camera direction, typically capturing the lateral walls, base and apex of the heart.
- Morphological operations (opening and closing with a radius of 2) are applied to the selected submesh to smooth the regions and eliminate undesired fragments.
- A change of basis is applied to extract the co‐ordinates aligned with the z‐axis and a perpendicular x‐axis, followed by the application of the calibration transformation matrix to convert the real space co‐ordinates into pixel space.

For regions where data from multiple cameras overlap, the overlaid data is combined using a weighted average, where the weight corresponds to the component of each vertex's normal relative to the direction of each camera. For areas with limited overlap, such as the borders and the apex, as well as to smooth transitions between maps, the values where there is no projection were interpolated linearly with the neighbour vertex values, iteratively, to ensure uniformity and accuracy in the resulting optical maps. A key limitation of the silhouette‐based approach is its inability to capture concavities that are not visible in the object's outer contour. This means that any concave structures in a horizontal cross‐section of the heart are not reconstructed, resulting in a lack of such details in each horizontal slice of the 3‐D mesh. To assess the spatial resolution and accuracy of our model, we provide a textured 3‐D mesh based on camera images, which offers an intuitive way to evaluate fine details. The reconstructed mesh has an approximate spatial resolution of 0.14 ± 0.02 mm per vertex (without any subdivision method), with an average reprojection DSC (dice similarity coefficient) of 95.79% (*N* = 4). The reprojection can be verified in Fig. 2*E*. At the top, the real images of the heart are reprojected onto the 3‐D geometry. At the bottom, the red mesh on the right, generated with a lower resolution, is shown for illustrative purposes showing the level of detail that the model can capture and how well the projection aligns with the input images. The identification of the electrodes over the 3‐D heart is performed by manually finding the electrodes in the optical data, and then its respective vertex of the geometry mesh is found by using the geometry/optical data relationship computed in the previous step (Fig. 2*F*). Figure 2*G* elucidates a typical 3‐D projection of the rabbit heart. The 3‐D projection is overlaid with a wavefront propagation during the atrial depolarisation, ventricular depolarisation and atria repolarisation.

### Electrocardiographic imaging

ECGi enables the reconstruction of epicardial electrograms (rEGMs) from non‐invasive measurements. In this approach, epicardial potentials are estimated by solving the Laplace equation within the volume conductor formed by the torso‐tank, based on the measured torso potentials and the geometric correspondence between the 3‐D surfaces of the heart and torso (Salinet et al., 2021). Preprocessing of the tank signals starts with Laplacian interpolation to enhance spatial resolution from 60 to 218 electrodes, ensuring better alignment with the tank geometry (Oostendorp et al., 1989). Baseline drift is corrected using an adaptive cubic spline filter (Sörnmo & Laguna, 2005), followed by a sixth‐order Butterworth low‐pass filter with a cut‐off frequency of 30 Hz to attenuate high‐frequency noise. This cut‐off was chosen based on spectral analysis using the fast Fourier transform (FFT). To improve computational efficiency without compromising signal integrity, the signals are downsampled from 4000 to 200 Hz. Subsequently, principal component analysis is applied to extract the most relevant components (Semmlow & Griffel, 2014), providing a compact and meaningful representation for use in ECGi reconstruction.

ECGi pipeline involved discretising the heart and torso surfaces into triangular meshes, allowing for a linear model to define the relationship between potentials on both surfaces. This relationship is expressed through the transfer matrix (Rudy, 2013; Salinet et al., 2021), computed using the boundary element method, which incorporates both the geometric structure and electrophysiological characteristics of the volume conductor (Salinet et al., 2021). The inverse problem in electrocardiography is inherently ill‐posed, meaning that small errors in torso potential measurements or geometry can lead to large inaccuracies in the reconstructed epicardial potentials (Cluitmans et al., 2015; Rudy, 2013). To address this instability, the inverse solution was obtained using zero‐order Tikhonov regularisation, currently regarded as the gold standard in ECGi (Tikhonov & Arsenin, 1977). The optimal regularisation parameter (λ) was selected using the L‐curve method, which balances fidelity and smoothness to minimise reconstruction error (Hansen & O'Leary, 1993).

### Cardiac mapping

#### Signal pre‐processing and filtering

Torso‐tank signals and EGMs had their baseline wandering removed through a high‐pass filter with a cut‐off frequency of 0.5 Hz. That was followed by a low‐pass Butterworth filter with a cut‐off of 250 Hz. Then, when present, 60 Hz and its harmonics were filtered using a notch filter (fourth order) with a quality factor of 80, a correction factor of 1.4 and a stop‐band attenuation of 120. Optical signals underwent baseline wandering removal using a high‐pass Butterworth filter with a cut‐off frequency of 0.5 Hz. Then, the signals were filtered with a spatio‐temporal Gaussian smoothing filter of 5 × 5 spatial filter kernel size and 1 × 7 temporal filter kernel size. The normalised change in fluorescence intensity (∆*F*/*F*) was calculated by (*F* − *F*_0_)/*F*_0_, where *F*_0_ represents the fluorescence intensity of the polarised cell's membrane and *F* of a depolarised cell's membrane (Uzelac et al., 2022), and presented in percentage. A binning factor of 8 was applied to the original optical data prior to filtering. This resulted in spatial averaging of pixel intensities, blending the electrode regions with the surrounding optical signals. Although this introduces some inherent noise near the electrodes, it ensures that optical data remain available across the field of view. Each electrode has a physical size of 1 × 1 mm, which, after binning, corresponds to ∼196 pixels arranged in a 14 × 14 grid. For EGM–optical signal comparison, optical traces were selected from regions located at least seven pixels (∼0.5 mm) away from the electrode edges. For signal characterisation, original optical signals were resampled from the original sample frequency of 500–4000 Hz, using the MATLAB (MathWorks, Natick, MA, USA) function ‘resample’ for linear interpolation.

#### Feature extraction and map generation

- **Local activation time and cycle length**: The local activation time (LAT) map in the torso‐tank signals was identified as +dV/dt (Brown et al., 2007) and −dV/dt for EGMs (Cantwell et al., 2015). The optical LAT is calculated by linear fit (Polyfit Matlab function) along the optical action potential (OAP) upstroke and determining the 50% rise of the OAP upstroke (Efimov et al., 2004; Fedorov et al., 2009). To generate the maps the ‘Imagesc’ MATLAB function was used where the colour bar is set between 5% and 95%. The cycle length (CL) for optical and electrical signals was calculated based on data from 16 specific points, each corresponding to an electrode location. For each electrode, a single pixel was selected from the surrounding neighbourhood of seven pixels closest to the electrode position. For each point, a time window containing 10 consecutive activations was chosen, and the mean CL was computed. The final reported CL value represents the average of the measurements from all 16 positions.
- **Dominant frequency**: Torso‐tank, EGM, rEGM and optical signals were first divided into segments of 4 s. For each signal, FFT was performed to estimate the frequency spectrum. A Hamming window and a zero‐padding factor of 5 were applied when performing the FFT, resulting in a frequency step of 0.05 Hz. Dominant frequency (DF) was defined as the most powerful frequency peak in the power spectrum. The organisation index was also calculated (Salinet et al., 2014, 2013).
- **Phase**: Hilbert transform *h*(*t*) was used to generate an analytic signal *F*(*t*), from which the instantaneous phase ϕ(*t*) of the non‐contact, epicardial contact EGMs and optical signals can be calculated as the four‐quadrant inverse tangent of the ratio of the imaginary *h*(*t*) and real part *f*(*t*) of the analytic signal [Eqns (5) and (6)], where *h*(*t*) is the Hilbert transform of the original signal *f*(*t*) (Salinet et al., 2015).
(5) $$F\;\left(t\right)=f\;\left(t\right)+jh\left(t\right)=A\left(t\right)e^{j\phi \left(t\right)}$$
(6) $$\phi \left(t\right)=tan^{-1}\left[\frac{h\left(t\right)}{f\left(t\right)}\right]$$

To create 2‐D maps from MEAs placed over the heart epicardium and electrodes around the hexagonal tank, we use flat surface meshes to model these structures for epicardial and non‐invasive electrical mapping, respectively. The MEAs are structured as a square mesh with 221 points (vertices) and 400 connections (faces), whereas the tank is represented as a rectangular mesh with 1201 points and 2304 connections. Because we only have recorded data from a subset of these points (16 for the MEAs and 60 for the tank), we use mathematical interpolation to estimate values across the entire surface. This interpolation is performed using Laplacian interpolation, which involves calculating a Laplacian matrix that captures how each point on the mesh is connected to its neighbours. First, the matrix is computed by iteratively determining edge lengths and populating a connectivity matrix. Then, we estimate the values for the remaining points on the surface using the Laplacian matrix and the known data points. By rearranging the Laplacian matrix to distinguish between known and unknown points, we reformulate the problem as a sparse linear system. Solving this system allows us to fill in the missing values and generate high‐density electrophysiological maps that cover the entire surface. Cross‐correlation (CC) is used to assess signal morphology similarity between EGMs and rEGMs because it quantifies shape agreement independently of amplitude differences (Cluitmans et al., 2018). To ensure consistency, both signals were normalised prior to analysis (Wang et al., 2021). A moving mean filter was applied to the rEGM signals to reduce artefacts and enhance activation clarity. Electrode positions were localised using optical mapping and the corresponding nodes on the 3‐D heart geometry were identified, enabling direct spatial comparison of EGM and rEGM signals.

## Results

The results presented below aim to illustrate the potential analyses that can be performed using the implemented platform, through the combination of its various components.

### Panoramic optical and electrical epicardium mapping

**Figure 3:**
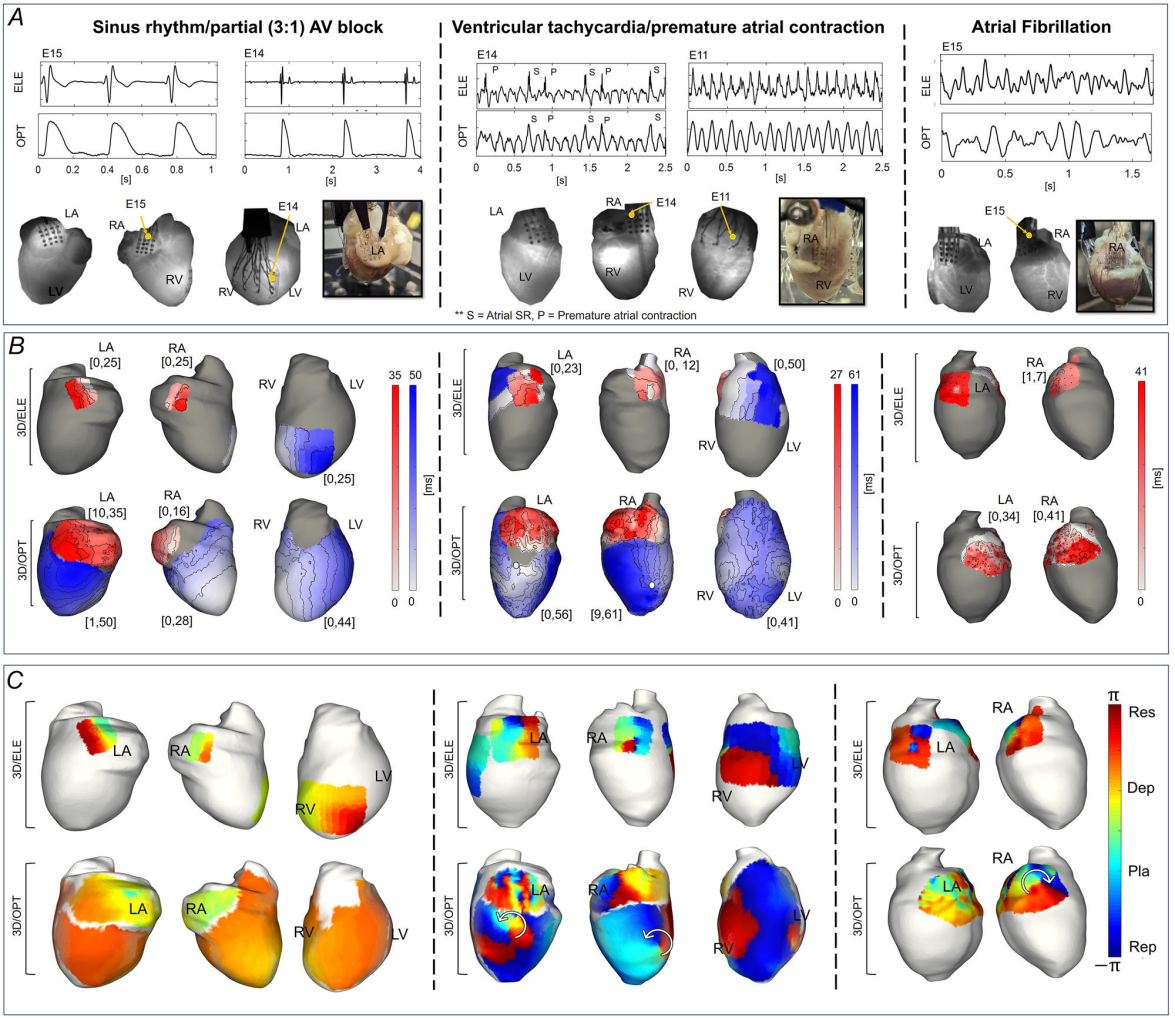
Simultaneous optical and invasive electrical mapping during sinus rhythm with AV block (SR/AVB), ventricular tachycardia with a premature atrial contraction (VT/PAC) and atrial fibrillation with AV block (AF) *A*, upper: showing representative optical and EGM signals recorded from selected regions on the heart surface, illustrating cardiac activity during SR/AVB (left), VT/PAC (middle) and AF (right). Lower: yellow arrows and circles indicate the anatomical locations where these signals were acquired, corresponding to the electrode positions shown in the camera‐acquired images. *B*, 3‐D LAT maps for electrical (top) and optical (bottom) data during SR/AVB (left), VT/PAC (middle) and AF (right). This illustrates the synchronisation of data capture and the differentiation between atrial (red) and ventricular (blue) activity. All isochronal lines in LAT are 5 ms apart, except AF with 2 ms. *C*, 3‐D phase maps for electrical (top) and optical (bottom) data under SR/AVB (left), VT/PAC (middle) and AF (right).

**Figure 4:**
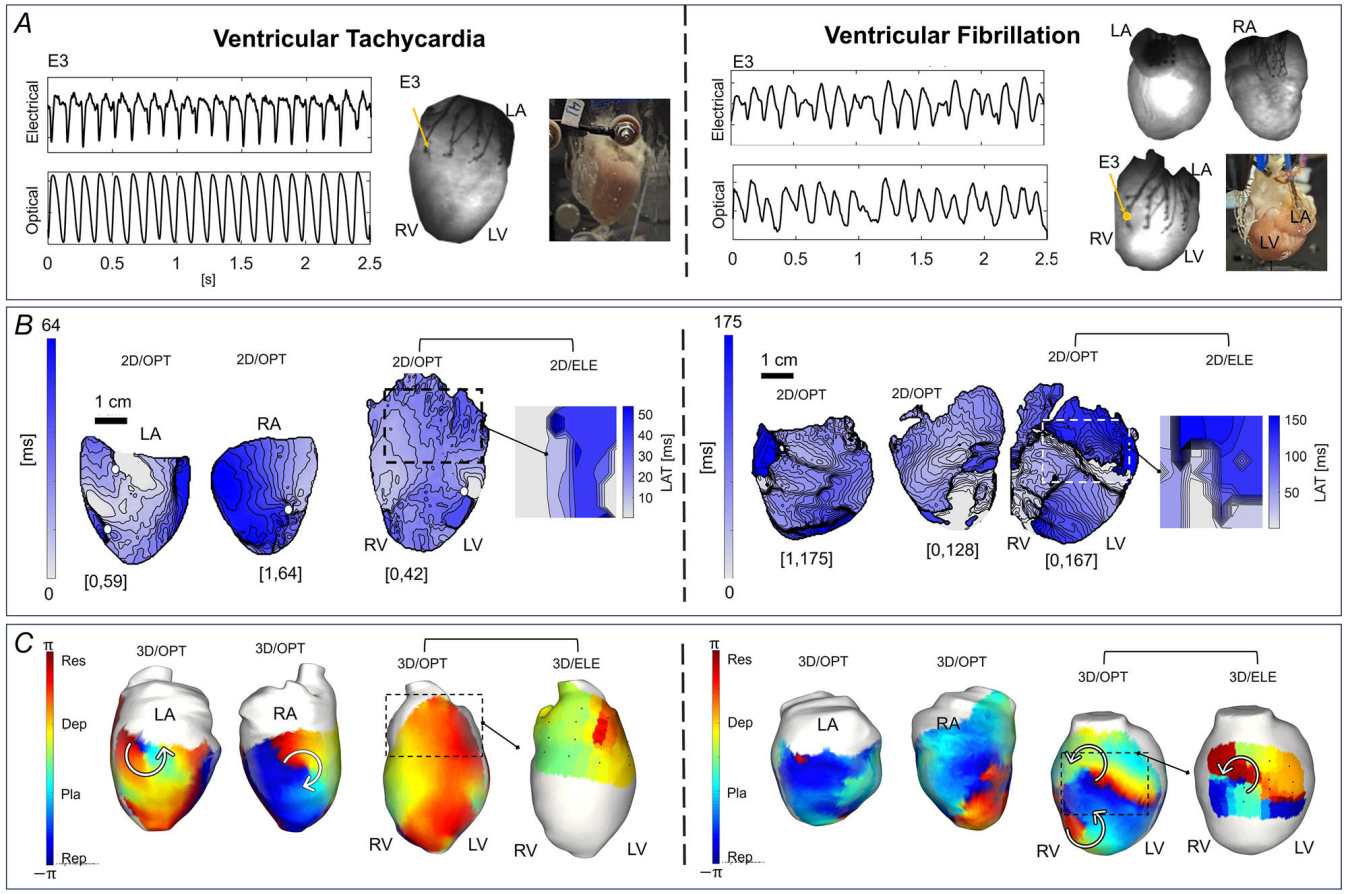
Comparative analysis of VT and VF *A*, electrophysiological recordings and anatomical context. Left: electrode 3 (E3) voltage signals and fractional change in fluorescence over time during VT, with anatomical position marked on the camera‐acquired images. Right: electrode 3 (E3) voltage signals and fractional change in voltage over time during VF, with electrode position indicated on the camera‐acquired images. Locations where the signals were obtained are highlighted with a yellow arrow and circle, corresponding to the electrode positions visualised in multi‐angle camera‐acquired images. *B*, optical LAT maps acquired from the three camera views, and contact electrical LAT map from the ventricular MEA, all isochronal lines in LAT are 5 ms apart. *C*, 3‐D optical phase maps acquired from the three camera views, and a 3‐D contact electrical phase map from the ventricular MEA.

The feasibility of the system to simultaneously map the epicardium using optical and electrical MEA mapping under different heart rhythms is presented in Figs 3 and 4. Figure 3*A* (upper) shows an optical signal and its respective EGM from the atrium and ventricle during sinus rhythm with AV block (SR/AVB), ventricular tachycardia with a premature atrial contraction (VT/PAC) and atrial fibrillation with AV block (AF). The respective images taken during the experiment are also shown. Heart's images demonstrate the strategic placement of the three MEAs used in the experiment but also underscore their crucial role in optimising both optical and EGM signal capture without mutual interference. Figure 3*B*, shows 3‐D LAT maps for electrical (top) and optical (bottom), for each of the three rhythms. The maps use separate colour schemes for atrial (red) and ventricular (blue) activities, offering insights into the timing and sequence of cardiac electrical activation. Additionally, the phase maps (Fig. 3*C*) provide a dynamic representation of rhythmic patterns, comparing EGM and optical maps side by side.

Figure 3*A* presents optical and electrical signals for one electrode over the RA (E15) and the ventricle (E14) during SR/AVB (left). Optical CL for RA was 370.1 ± 2.8 ms and 370.4 ± 0.2 ms for EGMs, whereas the ventricle presented 1442.1 ± 3.0 ms and 1442.2 ± 1.2 ms for optical and electrical data, respectively (E14). Results are similar, as expected between both modalities. The atria and ventricles are electrically isolated for the LAT map of SR (Fig. 3*B*), and the distance between isochrone lines is 5 ms for both atria and ventricle maps. In this scenario, optical maps display a travelling wave from the RA to the LA, depicted as red tones, and from the apex to the base for ventricular activity, shown as blue tones. Electrical mapping reveals the same pattern across the three MEAs. This expected propagation is further illustrated in optical and electrical data phase maps (Fig. 3*C*, left). In these maps, the RA is already depolarised, transitioning to the plateau phase, whereas the LA is just beginning to depolarise. Conversely, depolarisation in the ventricles initiates at the apex, spreading to the right and left ventricles. A similar behaviour is partially observed in the MEAs. The middle column of Fig. 3*A* shows a VT/PAC episode. The RA exhibits a slow SR with a CL of 556.4 ± 87.8 ms and 575.9 ± 43.1 ms for the optical and electrical data, respectively. The PAC activations (labelled ‘P’ in the plot) occurs following every other atrial sinus activation (labelled ‘S’), with an interval of 243.2 ± 25.7 ms in the optical signals and 215.9 ± 3.9 ms in the electrical signals, showing a certain difference between the mapping's modalities. Meanwhile, the ventricle displays episodes of VT, with a CL of 123.0 ± 9.3 ms for the optical signals and 127.2 ± 21.7 ms for the EGMs, sustained over a 2.5 s period. Because AV block is not present in this case, far‐field effects of ventricular activity are observable in the atrial signal. RA and LA show atypical propagation stemming from the supra‐ventricular ectopic beat, whereas RV and LV display re‐entrant waves, detected in both LAT and phase maps. LAT and phase maps for both atrial and ventricular regions were generated at different time points to capture the representative dynamics of the arrhythmia. The 3‐D LAT map shows the RA and LA are activated in 27 and 23 ms, respectively, with the earliest depolarised areas located in both atria (Fig. 3*B*, middle). The LA EGM and optical maps exhibit a similar wavefront behaviour, except for the upper right side with values around 40 ms presented in the electrical map. The LV harbours the earliest activation depolarisation area, which triggers the wavefront that travels to the two ventricles. The phase map shows two re‐entries (Fig. 3*C*, middle), one in each ventricle (white arrows), circulating in anticlockwise.

The third column shows atrial activity during an AF episode with AV block. RA activity CL is recorded at 151.4 ± 31.8 ms (6.9 ± 1.4 Hz) and 168.3 ± 57.4 ms (6.3 ± 1.2) Hz for optical and electrical, respectively. Fibrillatory activity is confirmed in both the RA and LA, as evidenced by the optical LAT and phase maps (Fig. 3*B* and *C*, right). There is absence of coherent wavefront propagation and independent activating re‐entrant waves in both LA and RA. As expected, the fibrillatory pattern shown with optical cameras could not be detected using the MEAs as a result of the MEA position over the atrial area. Figure 4 provides detailed visualisations of ventricular arrhythmias, VT on the right and ventricular fibrillation (VF) on the left. Figure 4*A* (left) presents optical and EGM signals corresponding to the experiment presented in Fig. 3 (VT/PAC), focusing only on the VT episode. In this case, a different electrode (E3, yellow point) presents a regular VT compared with E11 (Fig. 3*A* middle, yellow point), where both optical and EGM signals present a more irregular behaviour. Figure 4*A* (left) shows a stable re‐entrant activity over the LV, also confirmed in the respective LAT map, but with a slightly spatial localisation of the phase singularity (Fig. 4*B* and *C*, left). In VF, the HR is 349.5 ± 183.0 bpm in optical recordings and 526.4 ± 227.0 bpm in EGM signals showing discrepancies between mapping modalities. These LAT maps (Fig. 4*B*) reveal intricate re‐entrant patterns across the heart, also observed in phase maps (Fig. 4*C*). Videos are available demonstrating the temporal evolution of the optical signals on the mesh during SR/AVB (see Supporting information, Video S1) and VF (see Supporting information, Video S2).

### Non‐invasive electrical mapping (torso‐tank) and ECGi

**Figure 5:**
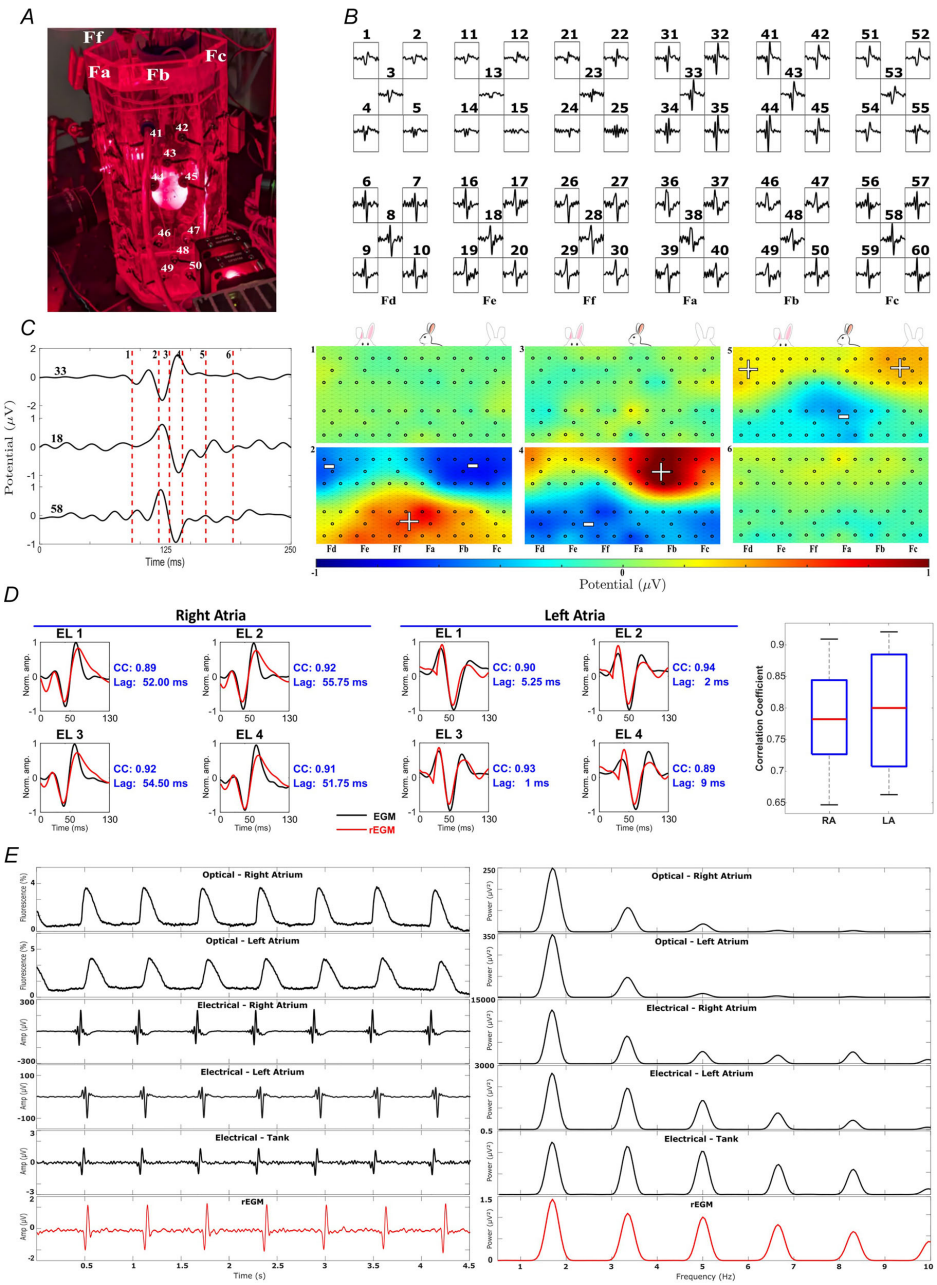
Non‐invasive mapping of a SR with AV node blockage *A*, torso‐tank setup with labelled faces and electrodes; the heart remains centrally positioned throughout the experiment. *B*, torso‐tank signals during SR, with electrode numbers and tank face labels (Fa–Ff) matching their positions. *C*, 250 ms segment from electrodes 33, 18 and 58 during SR (left); six red lines mark time points for torso‐tank potential maps (right), where white ± signals indicate relative potential levels. *D*, CC between EGMs (black) and rEGMs (red). Left and middle: representative 130 ms segments from four RA and four LA electrodes, with signals overlaid and aligned by computed lag; CC values and lags are shown. Right: boxplots of CC across all 16 electrodes per region. All signals normalised between –1 and 1. *E*, left: 4.5 s segment showing, from top to bottom, optical (RA, LA), electrical (RA, LA, tank) and reconstructed (rEGM) signals. Right: corresponding frequency spectra.

Figure 5*A* presents the torso‐tank model during an experiment with the heart in the centre. It can be observed the electrodes distribution on its faces and an area without electrodes, located in front of the optical cameras, to allow acquisition of the optical signals simultaneously. The white text labels illustrate the tank's faces and the corresponding number of electrodes. Figure 5*B* shows a single atrial beat during SR/AVB, recorded from the 60 tank electrodes. This visualisation demonstrates how activation is captured according to the electrode positions, with opposite activation directions between the upper and lower regions of the tank. In Fig. 5*C*, a 250 ms segment of the sinus signal from electrodes 33, 18 and 58 of the torso‐tank is shown. The electrode numbers follow the same pattern shown in Fig. 5*B*. Red dashed lines mark six different time points and, on the right side, potential maps of the torso‐tank corresponding to these points are plotted. Dashed trace 1 highlights the moment before activation starts, represented by an amplitude close to zero all over the torso‐tank, as shown in the respective potential map. Trace 2 shows the instant of the activation, where a higher potential is observed at the inferior half of the potential map, and a negative potential at the upper part, reflecting activations between sinoatrial and AV nodes. Positive potentials reflect the signal is travelling towards the electrode, whereas negative potentials indicate the potential moving away from the electrode. At this point, we can see that the potential is higher in the left frontal region of the torso‐tank. Trace 3 points a moment after the activation, when the potentials change polarity in the torso‐tank's upper and bottom parts, resulting in a map close to 0. Trace 4 points to the last part of the activation, represented by a more positive amplitude in the upper region and negative in the bottom, reflecting activations through atrial syncytial tissue and showing high potential in the upper left and back part of the torso‐tank surface. Trace 5 highlights a moment after activation, reflected as a more negative amplitude in the central region and slightly more positive at the top. Lastly, trace 6 shows the baseline, corresponding to a potential of near zero in the torso‐tank surface shown in the respective potential map.

ECGi analysis is summarised in Fig. 5*D* for SR. Figure 5*D* (left and middle) displays four representative electrodes from different regions from the RA and LA, showing segments 130 ms long, with EGM (black) and rEGM (red) signals overlaid and temporally aligned according to the computed lag. In the RA, electrodes EL2 and EL3 exhibited the highest CC values (0.92), although these were associated with the longest delays (lags around 55 ms). Across all 16 electrodes, lags consistently ranged from 45 to 56 ms, indicating a systematic delay in the reconstructed activation. In contrast, the LA demonstrated better temporal alignment, with lags not exceeding 9 ms. The highest correlation was 0.94 in EL2, with a minimal delay (2 ms), indicating strong morphological and temporal agreement. The boxplot on the right summarises the CC distribution across all electrodes in each region, highlighting slightly greater correlation performance in the LA. An overall mean CC was calculated as the average of 16 signals from the RA and LA, resulting in 0.79 ± 0.09 for the LA and 0.78 ± 0.08 for the RA during SR/AVB in a single beat.

Figure 5*E* (left) shows a 4.5 s segment of the optical signal, EGM, torso‐tank and rEGM. The CL between consecutive activations (600 ms) agrees across all mappings (optical, EGMs, torso‐tank and rEGM signals), corresponding to an activation frequency of 1.67 Hz, calculated as the inverse of the CL. On the right, the respective power spectra of each signal are shown. The DF and its harmonics match between the optical, EGMs, torso‐tank and rEGM signals. The DF is 1.68 Hz and agrees with the frequency of activation obtained by the CL. The analysis was extended for sinus tachycardia showing an agreement (DF: 4.8 ± 0.2 Hz) between the optical, EGM, tank‐torso and rEGM highlighting an agreement between the measured systems.

**Figure 6:**
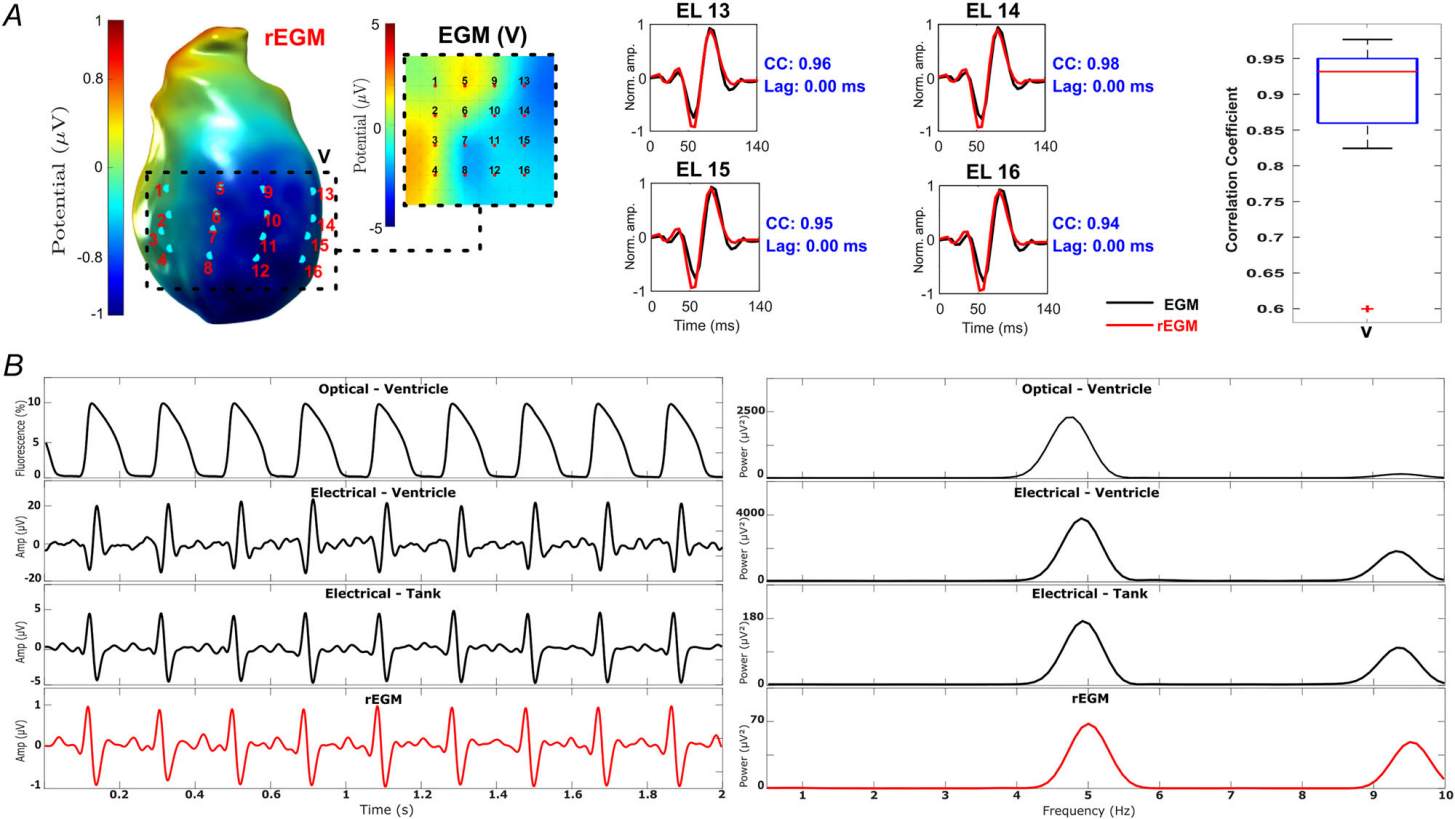
ECGi analysis during VT *A*, left: three‐dimensional rEGM potential map with highlighting ventricular electrode positions, alongside the corresponding two‐dimensional EGM potential map. Four representative electrodes (EL13–16) are shown with normalised EGM (black) and rEGM (red) signals overlaid, including their CC and lag values. Right: boxplot of CC values across the 16 ventricular electrodes. *B*, left: two‐second signal segments recorded from the ventricular region, showing optical fluorescence, measured EGMs, torso‐tank signals and the reconstructed rEGM. Right: frequency spectra of each signal.

A ECGi analysis was also conducted during VT, as shown in Fig. 6. The 3‐D potential map of the rEGM (Fig. 6*A*) illustrates a ventricular activation pattern, with the right half of the ventricle displaying more negative potentials, consistent with the wavefront originating from the right side. This spatial pattern agrees with the measured EGM potential map. However, a temporal mismatch is evident in the left region: electrodes 5–8 are already activated in the rEGM, whereas the EGM signals at electrodes 5 and 6 are still positive, indicating delayed activation in the measured data. Electrodes 1–4 on the left side also show higher magnitude in the EGM rather than in the rEGM. CC analysis was performed for electrodes located on the right side (electrodes 13–16), where the agreement was the highest. The maximum CC reached 0.98, and no temporal lag was observed at these electrodes, indicating very high morphological and temporal similarity. However, as shown in the potential maps, the left side of the ventricle exhibited temporal misalignment. In the rEGM, the activation appears nearly simultaneously across this region, whereas the EGM reflects a more gradual wavefront propagation.

## Discussion

In the present study, we introduce a novel platform that integrates panoramic optical and electrical mapping techniques, validated in isolated rabbit hearts. This platform represents one of the first high‐resolution, tri‐modal mapping applications in Langendorff‐perfused rabbit hearts, offering a tool for studying cardiac electrophysiology through both electrical and optical mapping. The complementary strengths of these techniques provide a comprehensive view of the heart's electrical activity, from the propagation of cellular APs to large‐scale conduction patterns that influence overall cardiac rhythm and function. Additionally, our study platform incorporates a torso‐tank model equipped with 60 electrodes for non‐invasive mapping, enhancing the translation relevance to clinical applications.

The initial analysis for setup validation confirms the feasibility of simultaneous optical and electrical mapping during sinus rhythm, as well as atrial and ventricular arrhythmias, using time, frequency and phase analyses. During sinus rhythm and ventricular tachycardia, wavefront propagation showed concordance between mapping modalities. However, because atrial arrhythmias revealed increased spatiotemporal complexity, diverges between the mapping strategies are present. DF from signals from the torso‐tank, optical recordings, EGM and rEGM agreed. In addition, EGM and rEGM presented a high correlation.

Optical mapping imaging on *ex vivo* perfused hearts has been instrumental in advancing our understanding of the mechanisms underlying cardiac arrhythmias in basic science research (Barone et al., 2020; Cherry & Fenton, 2008; Davidenko et al., 1992; Grey et al., 1998; Pastore et al., 1999; Uzelac et al., 2017; Watanabe et al., 2001). This progress has been supported by continuous advancements in optical mapping methodologies (Christoph & Luther, 2018; Lee et al., 2011; Uzelac et al., 2022; Zhang et al., 2016) and the development of signal and image processing techniques for optical mapping measurements (Christoph et al., 2017; Laughner et al., 2012; Pollnow, 2018; Tomek et al., 2021; Uzelac & Fenton, 2015). These primarily basic research efforts have provided valuable insights into arrhythmic mechanisms. Simultaneously, understanding and controlling arrhythmia mechanisms using optical mapping as a ‘visual aid’ has been an ongoing focus of research (Christini et al., 2006; Entcheva et al., 1998; Ji et al., 2017; Luther et al., 2011; Uzelac & Fenton, 2020; Wikswo et al., 1995). This presents complex, multidisciplinary challenges that require the integration of electrophysiology, biomedical engineering and computational modelling.

Because our platform is built upon a foundation of panoramic optical mapping, it is essential to review previous work in this area and highlight how our approach differs. Previous studies extensively employed panoramic optical mapping to investigate the spatial and temporal dynamics of cardiac arrhythmias, particularly in Langendorff perfusion setups. Notable work by Efimov's group includes the 3‐D panoramic imaging of cardiac arrhythmias in rabbit hearts (Lou Ripplinger et al., 2008; Qu et al., 2007), low‐voltage cardioversion (Ripplinger et al., 2009) and the development of the open‐source RHYTHM toolkit for panoramic optical mapping (Gloschat et al., 2018).

One of the earliest implementations was developed by Bray et al. (2000), who utilised two planar, front‐surface mirrors to achieve panoramic optical mapping in rabbit hearts using a single CCD camera operating at 335 frames s^−1^. Although this three‐view approach was cost‐effective, it considerably reduced spatial resolution, yielding only 128 × 64 pixels per projection of the heart. Subsequently, Kay et al. (2004) extended this methodology to larger mammalian hearts, specifically porcine models, employing a single mirror and two CCD cameras to acquire four views at a frame rate of 250 frames s^−1^. Later, Qu et al. (2007) proposed the use of photodiode arrays (PDA) instead of CCD cameras, enhancing the system's temporal resolution by increasing the sampling frequency to 5000 frames s^−1^ in a three‐view system, with a spatial resolution of 769 pixels (using three 16 × 16 PDAs). Both Kay et al. (2004) and Qu et al. (2007) utilised higher spatial resolution cameras compared to Bray et al. (2000) for 3‐D heart reconstruction, requiring a more comprehensive calibration process. Finally, Lee et al. (2017) developed a low‐cost panoramic optical mapping system using USB 3.0 CMOS cameras, aiming to make optical mapping more accessible to research laboratories. The system implemented in this study operates three optical cameras at a sampling frequency of 500 Hz, which is sufficient for the acquisition of cardiac arrhythmias, with an original spatial resolution of 1600 × 1000 pixels. Depending on the specific study requirements, this resolution can be reduced using binning to facilitate processing or retained in its original form for specialised analyses. Additionally, the use of the same camera for both fluorescence imaging and 3‐D reconstruction eliminates the need for an extended calibration process, thereby streamlining data acquisition and analysis.

These studies underscore the critical role of high‐resolution optical mapping in understanding arrhythmogenic mechanisms. However, our approach integrates real‐time electrical, for a more comprehensive view of cardiac electrophysiology. The integration of electrical mapping into panoramic optical mapping setups is essential for providing a more comprehensive understanding of cardiac arrhythmias. Although optical mapping provides high‐resolution visualisation of cellular‐level activity and AP propagation, it measures transmembrane potentials, which are fundamentally different from the extracellular potentials captured by clinical tools such as ECG or body surface potential mapping (BSPM). This intrinsic difference not only makes direct comparisons challenging, but also highlights the complementary nature of these modalities.

Electrical mapping, widely used in clinical settings, is vital for translating insights gained through optical mapping into practical, patient‐focused applications, such as guiding ablation procedures. Berenfeld's group initially employed a panoramic optical mapping system alongside electrodes placed on the surface of a torso‐tank model, without direct electrode placement on the heart (Berenfeld et al., 2011). This setup was used to analyse DF and CL during AF in Langendorff‐perfused sheep hearts, with a key finding that DF correlated more accurately with optical mapping data than CL. In 2017, their focus shifted to BSPM. A study by Rodrigo et al. (2017), highlighted BSPM as a non‐invasive tool for identifying DF in AF patients to improve ablation precision, although this study did not incorporate optical mapping. Bear et al. (2018) conducted a study using Langendorff‐perfused pig hearts to investigate electrical desynchrony through both invasive and non‐invasive electrical mapping techniques. They utilised an epicardial sock with 108 electrodes for invasive mapping and 128 electrodes embedded in a torso‐shaped tank for non‐invasive measurements, along with ECGi for reconstructing epicardial electrograms. By contrast, the present study focuses on smaller rabbit hearts and employs a different experimental configuration. Specifically, three MEAs comprising 16 electrodes each were positioned over targeted regions of interest, while 64 electrodes were distributed across the torso‐tank for non‐invasive mapping. As preliminary results from the proposed multimodal platform, we present optical and electrical maps in both 2‐D and as projections onto the reconstructed 3‐D heart geometry. In the 2‐D maps, potential differences in activation patterns between modalities (e.g. contact electrical and optical mapping), even after spatial alignment, may arise because of disparities in signal resolution, tissue–electrode interface quality or modality‐specific sensitivities to propagation dynamics. In the 3‐D projections, some spatial mismatches were observed, largely attributable to difficulties in distinguishing the MEAs from the tissue, subtle morphological changes during handling, and structural differences between the perfused and post‐perfusion states. Although an increase in heart volume was noted, consistent with previous electromechanical optical mapping studies (Bray et al., 2000; Gloschat et al., 2018; Kay et al., 2004; Lou Ripplinger et al., 2008; Qu et al., 2007), these changes were not accounted for in the current analysis. The final heart volume was used for reconstruction to minimise spatial distortions, and the rigid mounting helped contain expansion within physiological limits. Because the focus here is on the development and validation of the experimental platform, such deformations were not corrected, but future work will include enhanced post‐processing and dynamic volume tracking to improve spatial accuracy.

In the present study, the comparison between rEGMs and EGMs showed generally good agreement (typically above 0.7). A good match (defined as CC > 0.70) was observed in 78% of RA electrodes and 76% of LA electrodes. These values are comparable to previous findings in the literature. Cluitmans et al. (2017) reported a mean CC of 0.71 in anaesthetised dogs using body surface and epicardial recordings, while Ghosh and Rudy (2005) demonstrated a mean CC of 0.81 in torso‐tank experiments with explanted canine hearts over multiple beats. Under SR/AVB conditions, analysis of the LA revealed that some neighbouring electrodes exhibited distinct signal morphologies despite their spatial proximity. This local variability contributed to lower CC values (below 0.7) in certain electrodes, probably as a result of the limitations of the regularisation method, which struggles to capture abrupt spatial variations. Moreover, suboptimal contact between the MEA and the epicardial surface may have introduced non‐physiological distortions in the recorded signals, contributing to both reduced CC values and the lags consistently observed in the RA. The ‘graininess’ or spatial roughness observed in some rEGM maps may also reflect these limitations. This effect may arise from the use of zero‐order Tikhonov regularisation, which minimises signal magnitude without promoting spatial smoothness. In addition, noise in the torso‐tank signals and anatomical variability may contribute to abrupt variations in the rEGM maps. Frequency analysis further demonstrated that the reconstruction preserved the frequency components of the signals compared to the optical, EGMs, and tank signals in both sinus AVB and sinus tachycardia recordings.

The combination of optical and electrical mapping is valuable because it leverages the high‐resolution, cellular‐level insights provided by optical mapping with the broader, clinically relevant data captured by electrical methods such as BSPM. Optical mapping excels in detailing the propagation of APs and arrhythmic events with high spatial resolution, whereas electrical mapping is essential for translating these insights into actionable clinical data for therapies. The lack of integration between these two powerful approaches in most studies limits the ability to provide a comprehensive view of cardiac arrhythmias. By combining them, researchers can gain a deeper understanding of arrhythmic mechanisms, from cellular dynamics to large‐scale conduction patterns, enhancing both basic research and its translation into clinical applications. The tri‐modality platform presented in the present study addresses this gap, offering a more holistic approach to studying cardiac electrophysiology and the mechanisms driving arrhythmias.

## Conclusions

The present study contributes to ongoing electrophysiology research by developing an animal model platform with 3‐D reconstruction, utilising both high‐density panoramic optical and electrical mapping (epicardial and torso‐tank) techniques in isolated rabbit hearts. Panoramic optical mapping and electrical measurements were employed to comprehensively analyse spatio‐temporal characteristics, where sometimes they do not agree, and the non‐invasive mapping demonstrated the feasibility of the platform for studying cardiac rhythms. This study represents a pioneering effort in applying high‐resolution, dual‐modality mapping to Langendorff‐perfused rabbit hearts, thereby offering novel insights into the electrophysiological foundations of arrhythmogenic behaviour. In addition to this, the incorporation of ECGi further expands the investigative potential of the platform, enabling a more comprehensive characterisation of cardiac activation from a non‐invasive perspective. Altogether, the proposed platform not only facilitates the study of atrial and ventricular arrhythmias, but also provides a robust tool for integrated analysis of cardiac behaviour, as supported by the preliminary results obtained with this platform.

### Study limitations

The number of experiments was limited, primarily aiming to demonstrate the feasibility of the experimental setup for studying arrhythmias. Not all forms of arrhythmias were explored, and the focus was mainly on presenting the possible combination of modalities to acquire data from both optical and electrical mapping systems. Additionally, the analysis of non‐invasive and ECGi frequency characteristics was restricted to regular rhythms. The placement of the MEAs occasionally led to misinterpretations because the arrays did not always align with the intended cardiac regions; MEAs designed for atrial mapping sometimes covered ventricular areas, potentially skewing the results. The isolated Langendorff‐perfused rabbit heart model, althoughg valuable for controlled studies, lacks the complex physiological interactions present in vivo, such as neural inputs and hormonal regulation. Moreover, 3‐D mapping was employed to illustrate preliminary results, demonstrating the potential of the platform. The post‐processing of signals from all modalities, including optical, electrical and ECGi, will be further refined and explored in future studies to enable more robust and detailed electrophysiological analyses.

## Additional information

## Competing interests

The authors declare that they have no competing interests.

## Author contributions

J.S.: acquisition, analysis and interpretation of the work, drafting the work, revising in critically for important intellectual content, final approval of the version to be published and agreement to be accountable for all aspects of the work. V.S., T.N., A.Q. and I.U.: acquisition, analysis and interpretation of the work, drafting the work and revising in critically for important intellectual content, final approval of the version to be published and agreement to be accountable for all aspects of the work. I.S.: acquisition, analysis and interpretation of the work, drafting the work, final approval of the version to be published and agreement to be accountable for all aspects of the work. Ó.B.P.: analysis and interpretation of the work, final approval of the version to be published and agreement to be accountable for all aspects of the work. J.S.: conception and design of the work, acquisition, analysis or interpretation of the work, drafting the work or revising in critically for important intellectual content, final approval of the version to be published and agreement to be accountable for all aspects of the work. All authors have approved the final version of the manuscript submitted for publication and agree to be accountable for all aspects of the work, ensuring that any questions related to the accuracy or integrity of any part of the work are properly investigated and resolved. Additionally, all individuals designated as authors meet the criteria for authorship, and all those who qualify for authorship are listed.

## Funding

This study is supported by grant no. 2018/25606‐2, São Paulo Research foundation (FAPESP) and CNPq INCT INTERAS. Tainan Neves was supported by Coordenacão de Aperfeiçoamento de Pessoal de Nível Superior – Brasil (CAPES) and is supported by FAPESP grant no. 2024/02521‐2. Jimena Siles and Angélica Quadros are supported by grants no. 2020/03601‐9 and 2023/06306‐6, respectively, FAPESP.

## Supporting information




Peer Review History

**Video S1**. The top row displays raw optical images of a rabbit heart during sinus rhythm with atrioventricular (AV) block. From left to right: anterior view showing the right and left ventricles (RV/LV); view of the RA and right ventricle (RV), as well as view of the left atrium (LA) and left ventricle (LV). Superimposed on each image is a colour‐coded representation of the normalised signal intensity, where 0 appears transparent and 1 is indicated by green for ventricular regions and dark yellow for atrial regions. The middle row shows representative atrial optical signals from selected points in each view, and the bottom row presents the corresponding ventricular signals. Red dots indicate the analysed time point presented in ms.

**Video S2**. The top row shows raw optical images of a rabbit heart during ventricular fibrillation (VF). From left to right: view of the left atrium (LA) and left ventricle (LV); view of the right atrium (RA) and right ventricle (RV); and anterior view showing both ventricles (RV/LV). Superimposed on each image is a colour‐coded representation of the normalised signal intensity, where 0 appears transparent and 1 is shown in green for ventricular tissue. The middle row is left blank because atrial signals were not analysed during this episode. The bottom row displays representative optical signals from a selected ventricular site in each view, illustrating the irregular and rapid electrical activity characteristic of ventricular fibrillation. Red dots mark the analysed time point presented in milliseconds.

## Data Availability

Raw data are available from the corresponding author upon reasonable request.
