# Supplementary material for: An integrated platform for 2‐D and 3‐D optical and electrical mapping of arrhythmias in Langendorff‐perfused rabbit hearts
Source: J Physiol. 2025 Jun 25;604(13):5200–20. doi: 10.1113/JP287815 (PMC13327769; doi:10.1113/JP287815)
Supplement: Supplementary file 1 — Peer Review History [file TJP-604-5200-s001.pdf]

The Journal of Physiology JP

An Integrated Platform for 2D and 3D Optical and Electrical Mapping of Arrhythmias in Langendorff-Perfused Rabbit Hearts

Jimena Gabriela Siles Paredes, Vinícius Silva, Tainan Neves, Italo Sandoval, Angélica Drielly Santos de Quadros, Giovanni Weber, Óscar Barquero, Ilija Uzelac, and João Salinet
DOI: 10.1113/JP287815

Corresponding author(s): João Salinet (joao.salinet@ufabc.edu.br)

The following individual(s) involved in review of this submission have agreed to reveal their identity: Callum Michael Zgierski-Johnston (Referee #2); Anand Ganesan (Referee #3)

Review Timeline:

Submission Date:	21-Nov-2024
Editorial Decision:	16-Jan-2025
Revision Received:	13-May-2025
Accepted:	29-May-2025

Senior Editor: Bjorn Knollmann

Reviewing Editor: Axel Loewe

Transaction Report:

(Note: With the exception of the correction of typographical or spelling errors that could be a source of ambiguity, letters and reports are not edited. Depending on transfer agreements, referee reports obtained elsewhere may or may not be included in this compilation. Referee reports are anonymous unless the Referee chooses to sign their reports.)

Dear Dr Salinet,

Re: JP-RP-2024-287815 "(Integrated setup) Integrated Panoramic Optical and Electrical Mapping for Comprehensive Analysis of Cardiac Arrhythmias in Isolated Rabbit Hearts" by Jimena Gabriela Siles Paredes, Vinicius Silva, Tainan Neves, Italo Sandoval, Angélica Drielly Santos de Quadros, Giovanni Weber, Ilija Uzelac, and João Salinet

Thank you for submitting your manuscript to The Journal of Physiology. It has been assessed by a Reviewing Editor and by 3 expert referees and we are pleased to tell you that it is potentially acceptable for publication following satisfactory major revision.

Please advise your co-authors of this decision as soon as possible.

The referee reports are copied at the end of this email.

Please address all the points raised and incorporate all requested revisions or explain in your Response to Referees why a change has not been made. We hope you will find the comments helpful and that you will be able to return your revised manuscript within 2 months. If you require longer than this, please contact journal staff: jp@physoc.org. Please note that this letter does not constitute a guarantee for acceptance of your revised manuscript.

Your revised manuscript should be submitted online using the link in your Author Tasks: Link Not Available. This link is accessible via your account as Corresponding Author; it is not available to your co-authors. If this presents a problem, please contact journal staff (jp@physoc.org). Image files from the previous version are retained on the system. Please ensure you replace or remove any files that are being revised.

If you do not wish to submit a revised version of your manuscript, you must inform our journal staff (jp@physoc.org) or reply to this email to request withdrawal. Please note that a manuscript must be formally withdrawn from the peer review process at one journal before it may be submitted to another journal.

TRANSPARENT PEER REVIEW POLICY: To improve the transparency of its peer review process, The Journal of Physiology publishes online as supporting information the peer review history of all articles accepted for publication. Readers will have access to decision letters, including Editors' comments and referee reports, for each version of the manuscript, as well as any author responses to peer review comments. Referees can decide whether or not they wish to be named on the peer review history document.

ABSTRACT FIGURES: Authors are expected to use The Journal's premium BioRender account to create/redraw their Abstract Figures. Information on how to access this account is here: https://physoc.onlinelibrary.wiley.com/journal/14697793/biorender-access.

This will enable Authors to create and download high-resolution figures. If authors have used the free BioRender service, they can use the instructions provided in the link above to download a high-resolution version suitable for publication.

The link provided should only be used for the purposes of this submission. Authors will be charged for figures created on this account if they are not related to this manuscript submission.

LANGUAGE EDITING AND SUPPORT FOR PUBLICATION: If you would like help with English language editing, or other article preparation support, Wiley Editing Services offers expert help, including English Language Editing, as well as translation, manuscript formatting, and figure formatting at www.wileyauthors.com/eeo/preparation. You can also find resources for Preparing Your Article for general guidance about writing and preparing your manuscript at www.wileyauthors.com/eeo/prepresources.

RIGOUR AND REPRODUCIBILITY: We recommend authors consult The Journal's Rigour and Reproducibility requirements to ensure that their manuscript is suitable for publication: https://physoc.onlinelibrary.wiley.com/pb-assets/hub-assets/physoc/documents/TJP-Rigour-and-Reproducibility-Requirements.pdf

REVISION CHECKLIST:

Check that your Methods section conforms to journal policy: https://jp.msubmit.net/cgi-bin/main.plex?form_type=display_requirements#methods.

Check that data presented conforms to the statistics policy: https://jp.msubmit.net/cgi-bin/main.plex?form_type=display_requirements#statistics.

Upload a full Response to Referees file. To create your 'Response to Referees': copy all the reports, including any comments from the Senior and Reviewing Editors, into a Microsoft Word, or similar, file and respond to each point, using font or background colour to distinguish comments and responses and upload as the required file type.

Please upload two versions of your manuscript text: one with all relevant changes highlighted and one clean version with no changes tracked. The manuscript file should include all tables and figure legends, but each figure/graph should be uploaded as separate, high-resolution files.

You may also upload:

- 'Potential Cover Art' for consideration as the issue's cover image
- Appropriate Supporting Information (Video, audio or data set: see https://jp.msubmit.net/cgi-bin/main.plex?form_type=display_requirements#supp).

We look forward to receiving your revised submission.

If you have any queries, please reply to this email and we will be pleased to advise.

Yours sincerely,

Bjorn Knollmann
Senior Editor
The Journal of Physiology

REQUIRED ITEMS

- You must start the Methods section with a paragraph headed Ethical approval (https://jp.msubmit.net/cgi-bin/main.plex?form_type=display_requirements#methods).

Research must comply with The Journal's policies regarding animal experiments (https://physoc.onlinelibrary.wiley.com/hub/animal-experiments) and adherence to these policies must be stated in the manuscript.

Authors should confirm in their Methods section that their experiments were carried out according to the guidelines laid down by their institution's animal welfare committee, including an ethics approval reference number. The Methods section must contain a statement about access to food, water and housing, details of the anaesthetic regime: anaesthetic used, dose and route of administration, and method of killing the experimental animals.

- Please upload separate high-quality figure files via the submission form.

- Please ensure that any tables are editable and in Word format, and wherever possible, embedded in the article file itself.

- Please ensure that the Article File you upload is a Word file.

- Your paper contains Supporting Information of a type that we no longer publish, including supplementary tables and figures. Any information essential to an understanding of the paper must be included as part of the main manuscript and figures. The only Supporting Information that we publish are video and audio, 3D structures, program codes and large data files. Your revised paper will be returned to you if it does not adhere to our Supporting Information Guidelines.

- Please include an Abstract Figure file, as well as the Figure Legend text within the main article file. The Abstract Figure is a piece of artwork designed to give readers an immediate understanding of the research and should summarise the main conclusions. If possible, the image should be easily 'readable' from left to right or top to bottom. It should show the physiological relevance of the manuscript so readers can assess the importance and content of its findings. Abstract Figures should not merely recapitulate other figures in the manuscript. Please try to keep the diagram as simple as possible and without superfluous information that may distract from the main conclusion(s). Abstract Figures must be provided by authors no later than the revised manuscript stage and should be uploaded as a separate file during online submission labelled as File Type 'Abstract Figure'. Please also ensure that you include the figure legend in the main article file. All Abstract Figures should be created using BioRender. Authors should use The Journal's premium BioRender account to export high-resolution

images. Details on how to use and access the premium account are included as part of this email.

- Please include a full title page as part of your main article (Word) file, which should contain the following: title, authors, affiliations, corresponding author name and contact details, keywords, and running title.

EDITOR COMMENTS

Reviewing Editor:

Methods Details:
As pointed out by referee #3, further details would facilitate reproduction of this study.

Ethics Concerns:
Source of animals and access to food and water not currently described - please add this to the Methods.

Please also see 'Required Items' above.

Comments to the Author (Required):
This manuscript presents a combination of three methods: optical mapping, ECG imaging and multi-electrode mapping on Langendorff-perfused rabbit heart. This is a remarkable biomedical engineering achievement.

Being submitted as a "Research Article", the study falls short of my expectations in providing novel physiological insight. It might be better suited as a "Techniques for Physiology" article (see author guidelines for details).

Reviewers #1 and #2 point out a number of methodological issues and suggest additional analyses that would need to be addressed for potential consideration.

On top of the more detailed description of methods to facilitate reproduction and reuse suggested by reviewer #3, I would also encourage you to make the raw data available as recommended in the author guidelines: https://jp.msubmit.net/cgi-bin/main.plex?form_type=display_requirements#addinfo You are also invited to run for the Open Science Badge: https://jp.msubmit.net/cgi-bin/main.plex?form_type=display_requirements#opensciencebadges

I recommend further language editing (as an example, key point 5 is grammatically not correct).

Senior Editor:

I concur with the expert reviewing editor. Extensive revisions and rewriting as detailed by the reviewers would be required to make the work suitable for the journal. If you decide to revise the manuscript, please submit the manuscript as a "Techniques for Physiology" article and follow the author guidelines.

NOTE FROM EDITORIAL OFFICE: You will not be able to change the article type as part of the revision process. We have amended it at this end for you, in advance of the revised submission. Please see our Techniques for Physiology guidelines: https://jp.msubmit.net/cgi-bin/main.plex?form_type=display_requirements#techniques

If you have any questions about the article type, please let us know: jp@physoc.org

----

REFEREE COMMENTS

Referee #1:

Siles et al. present a ambitious platform for cardiac arrhythmia research, integrating panoramic optical mapping with epicardial and torso-tank electrical mapping in isolated rabbit hearts. The authors have clearly invested significant effort in

developing this integrated system, and the technical achievement of merging these modalities is indeed noteworthy. However, to fully realize the potential of this platform and enhance the impact of their work, some revisions are needed:

Major points:

1. How was the accuracy of the reconstructed 3D heart geometry mesh evaluated? In Figure 2F, there appear to be significant discrepancies between the mesh outline (red) and the actual heart shape in all 3 camera images. Is this the error for the final mesh, or an intermediate step in the refinement process? Furthermore, the rationale for using images from all three cameras for further mesh refinement (as implied in Figure 2F) is not entirely clear to me. Is the rotation, scale and position of the mesh not given by the camera calibration?

2. Are the intrinsic and extrinsic camera parameters (focal length, optical centers, rotation, translation, ...) computed for each camera from the calibration optical prism image data? Is the lens distortion corrected when projecting the camera images?

3. Given the complex geometry of the atria, particularly intricate folds and curvatures, how accurately does your 3D mesh reconstruction method capture these features? Please discuss the limitations of the silhouette-based approach for reconstructing geometrically complex structures. A mesh file with texture based on the camera images would be helpful to understand the spatial resolution and accuracy of the mesh. Additionally, could you give values for the spatial resolution of the mesh and provide visualizations or quantitative data of reprojection errors?

4. Optical mapping experiments with Blebbistatin can be susceptible to heart swelling, meaning an increase in the overall volume of the heart tissue over the course of the experiment. Have the authors observed any significant increases in the heart's volume or shape over time in their experiments? If so, do these changes influence the spatial mapping between optical and electrical data?

5. Figures 3A and 4A: Are the images of the heart rendered images of the mesh, or are they cropped images directly from the cameras? The sharp and clearly visible electrode wires suggest that these might be original camera images. However, this is not explicitly stated in the text and should be clarified. If the images are indeed rendered from the 3D mesh, please provide a description of the rendering process. Specifically, are the original camera images texture-mapped onto the mesh triangles, or are the mesh vertices assigned colors?

6. Figures 3A and 4A: The relationship between the optical traces and the electrode locations needs further clarification. Please specify precisely where the displayed optical traces are extracted from. Is it from the area immediately surrounding a specific electrode? Given that the electrodes are opaque and appear to block optical signals from the underlying tissue, it is unclear why the LAT and phase maps do not show any "blind spots" or missing data corresponding to the electrode locations. Please explain how the optical data is processed to avoid these blind spots. Are the optical traces extracted from the 3D mesh or raw camera images?

7. Figures 3B,C and Fig 4B,C: The optical and EGM maps show differences that are difficult to interpret due to their differing visualization geometries. To enable a more meaningful comparison, could the EGM data be mapped onto the reconstructed 3D mesh using the known electrode positions? Furthermore, what factors might contribute to any observed differences in activation patterns or values between the two modalities, even after spatial alignment?

8. A important consideration for the three-camera system is the extent of overlap between their respective fields of view and the resulting uniformity of signal quality across the epicardial surface. Do the three cameras provide sufficient overlap to capture every point on the heart's surface with a good viewing angle for accurate optical mapping? Are there regions, such as the apex or areas on the posterior or lateral walls, where the signal quality might be compromised due to a more oblique or tangential viewing angle from the cameras? In Figure 2H there appear to be areas near the apex where data is missing, and potential artifacts or discontinuities are visible at the transition zones between camera views.

9. Supplemental videos demonstrating the temporal evolution of the optical signals (or their phase) on the mesh would significantly enhance the manuscript. This would provide a dynamic visualization of the data and facilitate a more intuitive understanding of the spatiotemporal patterns.

10. In section 7.1: "RA presents a slow SR with a CL of 56.44{plus minus}87.82 ms (i.e to 109.22{plus minus}17.22 bpm) and 575.96{plus minus}43.16 ms (i.e to 104.53{plus minus}9.05 bpm) for optical and electrical data, respectively." The first CL value appears to be a misprint, it should be in the range of 600ms. In general, the precision with which the error values are reported throughout the manuscript is often excessive and not justified. I would suggest that the authors revise the number of significant digits used for reporting measured values and their errors to adhere to standard scientific practice.

11. In section 7.1 the optical CL values lack sufficient detail regarding the spatial and temporal averaging procedures. Please clarify over what spatial area the optical CL values are averaged. Is it over the entire field of view, specific regions of interest, or individual pixels? Over an entire recording?

12. The panoramic optical mapping data is not shown for the torso tank experiments, where the advantages of 3D mapping, such as spatial information across the entire epicardial surface, versus EGM validation would be most apparent. The optical data should be incorporated into the validation of the rEGM maps, e.g. with a supplementary video comparing the temporal evolution of activation patterns from both optical mapping and ECGI reconstructions.

13. The reconstructed electrogram maps in Figure 5D exhibits a degree of "graininess" or "roughness" that deviates from the smooth spatial gradients typically expected in electrophysiological signals. Could you comment on the factors that contribute to this? This seems to contribute the lag of the rEGM of electrode 16. The EGM/rEGM plots should also be plotted on top of each other so that they can be compared more directly.

14. The manuscript focuses on sinus rhythm to evaluate the rEGM signals, which has a very fast and spatially uniform activation pattern and does not reflect the complexity of clinically relevant arrhythmias. Validating ECGI during pacing or tachycardia, which are more representative of clinically important scenarios, would greatly enhance the impact of this manuscript.

15. The manuscript would benefit from a more explicit and detailed discussion of the advantages of panoramic optical mapping. Furthermore, a more thorough review of previous work in this area is needed, particularly regarding how the authors' approach differs from and potentially improves upon earlier methods. For example, the seminal studies by Bray et al. (2000) DOI: 10.1109/10.871412 or Kay et al. (2004) DOI: 10.1109/TBME.2004.827261 appear to be pertinent to this approach and should be mentioned or discussed.

Minor points:

1. The manuscript states that the 3D geometry is saved in five resolutions (20000, 10000, 5000, 2500, and 1200 vertices). What is the purpose of using different mesh resolutions? The manuscript does not clarify which resolution is used in the subsequent analysis.

2. How strongly is the mesh smoothed during the refinement? Could you perhaps show the difference between the raw and the refined mesh?

3. What is the bit depth of the camera images? 12-bit?

4. LAT abbreviation is used in 6.7 before it has been introduced.

5. The description of the ECGI reconstruction process could benefit from further detail to ensure the methodology is clear and reproducible.

6. Many journals names in the references are not correctly capitalized.

Referee #2:

The manuscript from Siles et al. describes a new system for simulataneously performing optical mapping, ECGi and multi-electrode mapping on a Langendorff-perfused rabbit heart. The approach is interesting and the description likely to be of benefit to the community. The manuscript would benefit from some additional analysis and further copy editing, but I have no major concerns.

The torso tank seems relatively small relative to the size of the heart. How does this compare to the relative size of heart and torso for humans and what effect will this have?

The extent of interpolation for both the MEA and surface tank is extremely large (nearly 14x for MEA and 20x for the surface tank). Is this valid? Can you compare with optical mapping or a recording done with more electrodes (even if not done simultaneously)?

The correlation of results seems very good between systems. It would be good to also examine a spatial correlation especially between ECGi and optical mapping. Perhaps something such as the similarity or nearness of phase singularity positions would be beneficial. It seems ECGi would smooth this and likely position further away, but that would perhaps highlight the advantage of this system in that you could quantify under what conditions ECGi or MEA can accurately track phase singularities.

Minor comments:

- The results section is a bit too descriptive, describing exactly what is happening in the figures rather than summarizing them.

- Fig. 5 - the numbering of electrode 16 does not fit with the general numbering and makes the example slightly confusing. Perhaps the numbering could be altered to be standardized or a different example face could be shown as all others number sequentially. Also in panel C showing a schematic of the heart rather than of a rabbit may help the readers to better orient themselves.

- In the discussion it is highlighted that optical mapping cannot capture electrical potentials the same way as clinical tools do. I think this should be rephrased slightly as it implies this is a disadvantage, whereas it's more just a symbol of the differences in the systems and optical mapping is better in that you see true transmembrane potential dynamics. I would instead just highlight that direct comparisons with clinical measurements is challenging as relating transmembrane potentials to extracellular potentials isn't necessarily straightforward.

Referee #3:

Paredes et al. provide the first description of a novel system developed by their group that allows the simultaneous acquisition of optical mapping, electrogram and ECGi based mapping in in vivo Langendorff style preparations. The paper is well-written, and substantially adds to the field by developing novel methods that are a major advance over existing tools. The methods are described well overall, and the techniques are a sufficient advance in my view to warrant a specific manuscript to describe them.

My only concern is whether there is sufficient detail to allow replication experiments. In particular, I think more detail or plannning diagrams would be helpful to allow the tank setup to be replicated. The paper of Lee et al 2017 cited by the authors is an example manuscript in the field that comparably aimed to allow other investigators to replicate the scientific experiments.

I think more detail will ultimately benefit the authors by allowing additional investigators to map atrial and ventricular arrhythmias as described by the authorship group.

END OF COMMENTS

EDITOR COMMENTS

Reviewing Editor:

1. Methods Details: As pointed out by referee #3, further details would facilitate reproduction of this study.

Answer: As pointed out by referee #3, further details have been provided to facilitate the reproduction of this study.

2. Ethics Concerns: Source of animals and access to food and water not currently described - please add this to the Methods.

Answer: The following paragraph was added to the methods section:

Methods section- Local Ethical Approval (Lines 121-124, page 4)

“This study was approved by the Ethics Committee on the Use of Animals of the Federal University of ABC in São Paulo, Brazil, under protocol 3947230519. All procedures were carried out according to institutional guidelines for animal experimentation. The rabbits, sourced from an authorized supplier (Granja RG), were kept in a light/dark cycle and were provided with balanced food supplied by the vendor. Access to fresh and clean drinking water was not restricted. In addition, the system featured air exhaust and continuous climate control (22 °C) throughout the pre-surgical period.”

Comments to the Author (Required):

3. This manuscript presents a combination of three methods: optical mapping, ECG imaging and multi-electrode mapping on Langendorff-perfused rabbit heart. This is a remarkable biomedical engineering achievement. Being submitted as a "Research Article", the study falls short of my expectations in providing novel physiological insight. It might be better suited as a "Techniques for Physiology" article (see author guidelines for details).

Answer: Thank you for your suggestion. The manuscript will be submitted as a Techniques for Physiology article. To align with the journal’s scope, the following modifications have been made.

Introduction section (Lines 104-115, page 4)

“The development of an integrated platform comprising the electrical mapping (invasive and non-invasive) and panoramic optical mapping would be of great importance to improve the current knowledge of cardiac arrhythmias and contributing to improve the current commercial system used to guide catheter ablation. In this study, we present an experimental setup that combines

simultaneously 3D panoramic optical mapping with multi-electrode epicardial electrical mapping and non-invasive electrical map- ping (torso-tank setup) for ECG imaging (ECGi) in Langendorff-perfused rabbit hearts. The combination of this multimapping modalities represents a significant technological advance in experimental cardiac electrophysiology by enabling a comprehensive view of cardiac electrical activity across multiple spatial scales and perspectives. This unique configuration allows for the direct comparison of cellular APs with surface EGMs and reconstructed ECGi signals, enabling mechanistic insights into the initiation and maintenance of arrhythmias, not achievable with existing techniques alone."

4. Reviewers #1 and #2 point out a number of methodological issues and suggest additional analyses that would need to be addressed for potential consideration.

Answer: Thank you for the constructive feedback. We have addressed the methodological concerns raised by Reviewers #1 and #2 and have also performed the recommended analyses. These revisions are highlighted in red in the manuscript, and the corresponding line numbers have been included in each reviewer comment for easy verification.

5. On top of the more detailed description of methods to facilitate reproduction and reuse suggested by reviewer #3, I would also encourage you to make the raw data available as recommended in the author guidelines: https://jp.msubmit.net/cgi-bin/main.plex?form_type=display_requirements#addinfo You are also invited to run for the Open Science Badge: https://jp.msubmit.net/cgi-bin/main.plex?form_type=display_requirements#opensciencebadges

Answer: Thank you for the suggestion. We have included a more detailed description of the methods as recommended by reviewer #3 to facilitate reproduction and reuse. Additionally, the raw data will be made available in accordance with the author guidelines. We also appreciate the opportunity to apply for the Open Science Badge and will ensure that all necessary steps are taken to meet the requirements.

6. I recommend further language editing (as an example, key point 5 is grammatically not correct).

Answer: Thank you for pointing this out. We have thoroughly reviewed and edited the language throughout the manuscript to ensure grammatical accuracy and clarity.

Key point (Line 26-29, page 1)

" A novel 3D platform that integrates panoramic and electrical mapping techniques (epicardium, non-invasive torso-tank and ECGi) is presented and validated in isolated rabbit hearts, highlighting that is not always that the mapping strategies agree, helping to further improve commercial systems. "

Senior Editor:

7. I concur with the expert reviewing editor. Extensive revisions and rewriting as detailed by the reviewers would be required to make the work suitable for the journal. If you decide to revise the manuscript, please submit the manuscript as a "Techniques for Physiology" article and follow the author guidelines.

Answer: The manuscript will be resubmitted as a *Techniques for Physiology* article, following the author guidelines and incorporating the necessary modifications to align with the journal's scope. In addition, the paper has been reviewed aiming improving the flow, technical descriptions and English.

8. NOTE FROM EDITORIAL OFFICE: You will not be able to change the article type as part of the revision process. We have amended it at this end for you, in advance of the revised submission. Please see our Techniques for Physiology guidelines: https://jp.msubmit.net/cgi-bin/main.plex?form_type=display_requirements#techniques If you have any questions about the article type, please let us know: jp@physoc.org

Answer: Thank you for amending the article type on your end. We acknowledge the update and have reviewed the *Techniques for Physiology* guidelines in preparation for the revised submission.

REFEREE COMMENTS

Referee #1:

Siles et al. present an ambitious platform for cardiac arrhythmia research, integrating panoramic optical mapping with epicardial and torso-tank electrical mapping in isolated rabbit hearts. The authors have clearly invested significant effort in developing this integrated system, and the technical achievement of merging these modalities is indeed noteworthy. However, to fully realize the potential of this platform and enhance the impact of their work, some revisions are needed:

Major points:

1. How was the accuracy of the reconstructed 3D heart geometry mesh evaluated? In Figure 2F, there appear to be significant discrepancies between the mesh outline (red) and the actual heart shape in all 3 camera images. Is this the error for the final mesh, or an intermediate step in the refinement process? Furthermore, the rationale for using images from all three cameras for further mesh refinement (as implied in Figure 2F) is not entirely clear to me. Is the rotation, scale and position of the mesh not given by the camera calibration?

Answer: Thank you for your thoughtful observation. We acknowledge the discrepancies in Figure 2F and appreciate the opportunity to clarify this aspect. For the first question "*How was the accuracy of the reconstructed 3D heart geometry mesh evaluated?*". To investigate the accuracy of the reconstructed geometry, we conducted a quantitative comparison between our in-house segmentation and an MRI-based segmentation (review purposes only and not for final publication), please see below:

Supporting information -3D Surface Validation through MRI (Line 1048-1078)

"For the validation of the 3D surface reconstruction, an MRI scan of the rabbit heart was acquired using a Siemens MAGNETOM 7 Tesla scanner. The heart was placed in a sample cup filled with Fomblin and stabilised with foam to maintain its position and prevent floating. The MRI data were segmented using 3D Slicer to obtain a high-resolution reference model of the heart (**Fig. 1A**). **Figure 1B** displays a photograph of the rabbit heart (left), the 3D surface generated using the developed system (middle), and the 3D model reconstructed from the MRI (right). Prior to comparison, a scale mismatch between the MRI segmentation and the in-house reconstruction was addressed: the MRI model was approximately ten times larger. This difference was corrected using Slicer's Transforms module, applying a scaling factor of 0.1, followed by translation and rotational alignment to ensure proper overlap between the two models. The Dice similarity coefficient was subsequently computed using Slicer's Segment Comparison module, with the MRI segmentation set as the reference and the in-house segmentation as the comparison model. The resulting Dice coefficient was approximately 0.74, indicating a moderate level of overlap between the two segmentations. This value reflects both the accuracy of the reconstruction and the inherent limitations of the silhouette-based method in

capturing internal concave structures. **Figure 1C** provides a visual representation of the reconstructed volumes, and their overlap as visualised in the Segment Comparison module. In this figure, the in-house volume is shown in light green, while the MRI-based segmentation appears in orange, highlighting regions of agreement and discrepancy. Overall, the analysis confirms that while the developed reconstruction technique provides an accurate external representation of the heart, it remains limited in its ability to capture internal concavities."

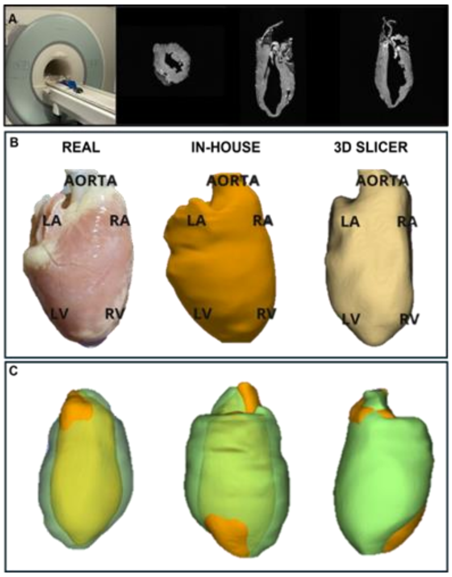

Figure 1 (not included in the paper): 3D Surface Validation through MRI. A. MRI acquisition using a 7T Siemens MAGNETOM scanner. **B.** Comparison between the real rabbit heart (left), the in-house segmented volume (middle), and the 3D Slicer segmented volume (right). The anatomical labels indicate the right atrium (RA), left atrium (LA), right ventricle (RV), and left ventricle (LV). **C.** Volume overlap analysis, the in-house segmented volume is shown in light green, and the MRI-based segmentation is shown in orange.

Regarding the second question "*In Figure 2F, there appear to be significant discrepancies between the mesh outline (red) and the actual heart shape in all 3 camera images. Is this the error for the final mesh, or an intermediate step in the refinement process?*".

Answer: Figure 2F, <u>now Figure 2D</u>, illustrates the alignment process of the final 3D heart geometry with the 2D optical images obtained from the three cameras during the experiment, which is performed offline for the study of cardiac electrical activity. Specifically, the 3D heart mesh generated in the previous reconstruction steps is projected onto each 2D camera view based on the known spatial orientation of each camera relative to the heart. Fine alignment is achieved through iterative rotation and translation of the 3D mesh to optimise its correspondence with the recorded images. The projection of the 3D geometry onto the 2D camera images follows a systematic approach: (i) Only the vertices of mesh triangles with a positive normal component in the direction of the respective camera are selected, ensuring that only surfaces facing the camera are visualised. The silhouette (highlighted in red in Figure 2D) is defined by triangles whose normals are nearly perpendicular to the camera direction, typically capturing the lateral walls, base, and apex of the heart. (ii) Morphological operations are applied to the selected sub mesh to smooth the regions and eliminate

undesired fragments. (iii) A change of basis is applied to extract the coordinates aligned with the Z-axis and a perpendicular Xi-axis, followed by the application of the calibration transformation matrix to convert the real space coordinates into pixel space. The discrepancies observed between the red mesh outline and the heart images in Figure 2D reflect the final alignment of the reconstructed 3D mesh rather than an intermediate step. These differences arise mainly from three factors:

- The challenge of distinguishing the microelectrode arrays (MEAs) from the heart tissue, as the MEAs extend beyond the mesh outline.
- Minor morphological changes introduced by handling the heart during the experiment.
- Structural differences between the heart's state during signal acquisition (when perfused) and during geometry reconstruction.

Therefore, the deviations seen in Figure 2D represent the final mesh projection and are inherent to the experimental and reconstruction conditions described.

Finally, regarding the last comment "*Furthermore, the rationale for using images from all three cameras for further mesh refinement (as implied in Figure 2F) is not entirely clear to me. Is the rotation, scale and position of the mesh not given by the camera calibration?*".

Answer: The camera calibration provides the necessary parameters for projecting the mesh vertices onto each camera image. While the rotation, scale, and position of the heart can be determined during the geometry step (at the end of the experiment), minor variations throughout the experiment may still occur. These discrepancies can reflect larger errors in the projected data but are mitigated through the additional refinement step using images from all three cameras.

We have addressed this point in the discussion and method section, where we state:

<u>Method Section - 3D heart geometry (Lines 311-325, page 11)</u>

> "**Figure 2D** illustrates the alignment process of the final 3D heart geometry with the 2D optical images obtained from the three cameras during the experiment. Specifically, the 3D heart mesh generated in the previous reconstruction steps is projected onto each 2D camera view based on the known spatial orientation of each camera relative to the heart. Fine alignment is achieved through iterative rotation and translation of the 3D mesh to optimise its correspondence with the recorded images. The projection of the 3D geometry onto the 2D camera images follows a systematic approach:
> (i) Only the vertices of mesh triangles with a positive normal component in the direction of the respective camera are selected, ensuring that only surfaces facing the camera are visualised. The silhouette (highlighted in red in **Fig. 2D**) is defined by triangles whose normals are nearly perpendicular to the camera direction, typically capturing the lateral walls, base, and apex of the heart.
> (ii) Morphological operations (opening and closing with a radius of 2) are applied to the selected sub mesh to smooth the regions and eliminate undesired fragments.
> (iii) A change of basis is applied to extract the coordinates aligned with the Z-axis and a perpendicular X-axis, followed by the application of the calibration transformation matrix to convert the real space

coordinates into pixel space."

Discussion section (Line 700-719, page 26)

"In contrast, the present study focuses on smaller rabbit hearts and employs a different experimental configuration. Specifically, three microelectrode arrays (MEAs) comprising 16 electrodes each were positioned over targeted regions of interest, while 64 electrodes were distributed across the torso tank for non-invasive mapping. As preliminary results from the proposed multimodal platform, we present optical and electrical maps in both 2D and as projections onto the reconstructed 3D heart geometry. In the 2D maps, potential differences in activation patterns between modalities (e.g., contact electrical and optical mapping), even after spatial alignment, may arise due to disparities in signal resolution, tissue–electrode interface quality, or modality-specific sensitivities to propagation dynamics. In the 3D projections, some spatial mismatches were observed, largely attributable to difficulties in distinguishing the microelectrode arrays from the tissue, subtle morphological changes during handling, and structural differences between the perfused and post-perfusion states. Although an increase in heart volume was noted, consistent with previous electromechanical optical mapping studies (Bray *et al.* 2000, Gloschat *et al.* 2018, Kay *et al.* 2004, Lou Ripplinger C. *et al.* 2008, Qu *et al.* 2007), these changes were not accounted for in the current analysis. The final heart volume was used for reconstruction to minimise spatial distortions, and the rigid mounting helped contain expansion within physiological limits. As the focus here is on the development and validation of the experimental platform, such deformations were not corrected, but future work will include enhanced post-processing and dynamic volume tracking to improve spatial accuracy."

2. Are the intrinsic and extrinsic camera parameters (focal length, optical centers, rotation, translation, ...) computed for each camera from the calibration optical prism image data? Is the lens distortion corrected when projecting the camera images?

Answer: The intrinsic camera parameters and the coordinates of each camera in the world reference frame have been added to the Methods section, within the description of the optical mapping setup. The extrinsic camera parameters were determined based on optical prism calibration and predefined values, and a clearer explanation was added to the Methods section under 3D heart geometry.

Methods section-Panoramic optical mapping (Lines 234-248, page 8-9)

"The intrinsic parameters also include a pixel size of 9×9 μm and a sensor size of 1.1" (17.6 mm CMOS). The lens has a focal length of 25 mm and a distortion smaller than 1% within the region of interest (ROI), which was considered negligible. Cameras were placed at a height of 9 cm above the base, positioned 120° apart around the tank. Their radial distance (R) from the tank centre varies between 20 cm and 24 cm, according to the heart's size, forming an annular arrangement instead of a fixed circular configuration. The coordinates for each camera in the world reference frame are expressed as follows:

$$Camera\ A: (R, 9, 0)\ , \text{with } 20\ \leq\ R\ \leq\ 24\ \text{cm} \tag{1}$$

$$Camera\ B\colon \left(-\frac{R_2}{2}, 9, \frac{\sqrt{3}R_2}{2}\right), \text{with } 20 \ \leq \ R \ \leq \ 24 \text{ cm} \tag{2}$$

$$Camera\ C\colon \left(-\frac{R_2}{2}, 9, -\frac{\sqrt{3}R_2}{2}\right), \text{with } 20 \ \leq \ R \ \leq \ 24 \text{ cm} \tag{3}$$

All three cameras visualised the ventricles, with additional coverage of the LA by Camera A and the RA by Camera B. Recordings were acquired simultaneously at 500 frames per second, with a resolution of 1600 × 1000 pixels, 12-bit depth, and a total duration of 10 seconds (yielding 5000 frames), using Norpix-StreamPix 9 software."

Methods section-3D heart geometry (Lines 264 – 279, pages 9-10)

"Extrinsic parameters of the camera were determined based on optical prism calibration and predefined values. The focal length was initially set to infinity, with plans for future computation. The optical centre was assumed to be the central pixel of the image. The extrinsic parameters, including the rotation matrix (R) and translation vector (t), were determined and incorporated into a similarity transformation matrix (M), that is, a transformation that combines rotation, dilation, and shift from origin. This matrix maps pixel coordinates to real-world space, with the origin set at the centre of the tank. ensuring proper alignment for image acquisition. The transformation from pixel space to real-world coordinates follows the equation:

$$P_w = MP_C \tag{4}$$

Where *Pw* denotes the real-world coordinates of a point and *Pc* the corresponding pixel coordinates, the transformation is defined by matrix *M*. This matrix is obtained by solving a linear system based on known prism landmarks and their pixel locations. Initially an affine transformation, *M* is regularised by removing shear components, resulting in a similarity transformation. Camera orientations are defined by inclination angles (α, β, γ) relative to the tank coordinate system. Since the cameras face the heart without vertical tilt, $\alpha = 0^\circ$. The angle β is determined by camera positioning: 0° for Camera A, 120° for Camera B, and 240° for Camera C. The roll angle is assumed to be $\gamma = 0^\circ$."

3. Given the complex geometry of the atria, particularly intricate folds and curvatures, how accurately does your 3D mesh reconstruction method capture these features? Please discuss the limitations of the silhouette-based approach for reconstructing geometrically complex structures. A mesh file with texture based on the camera images would be helpful to understand the spatial resolution and accuracy of the mesh. Additionally, could you give values for the spatial resolution of the mesh and provide visualizations or quantitative data of reprojection errors?

We appreciate the reviewer's observation. Assuming a reconstruction with 20,000 vertices covering an average area of approximately 2902 ± 400 mm^2, the average resolution per vertex is around 0.15 ± 0.2 mm^2, which corresponds to a linear spacing of approximately 0.54 ± 0.07 mm between vertices.

This clarification has been added to the Methods section – 3D heart geometry. Additionally, Figure 2 has also been modified to add the reprojection accuracy of the 3D mesh.

Methods section-3D heart geometry (Lines 307-311, page 11)

"**Figure 2C** shows three refined meshes, corresponding to each camera, obtained from the volume boundaries, after applying simplification and filters. Assuming a reconstruction with 20,000 vertices covering an average area of approximately 2902 ± 400 mm^2, the average resolution per vertex is around 0.15 ± 0.2 mm^2, which corresponds to a linear spacing of approximately 0.54 ± 0.07 mm between vertices."

Methods section-Figure 2 (Lines 335– 341, page 12)

"The reconstructed mesh has an approximate spatial resolution of 0.14 ± 0.02 mm per vertex (without any subdivision method), with an average reprojection DSC (dice similarity coefficient) of 95.79% (N=4). The reprojection can be verified in the **Figure 2E**. At the top, the real images of the heart are reprojected onto the 3D geometry. At the bottom, the red mesh on the right, generated with a lower resolution, is shown for illustrative purposes showing the level of detail that the model can capture and how well the projection aligns with the input images."

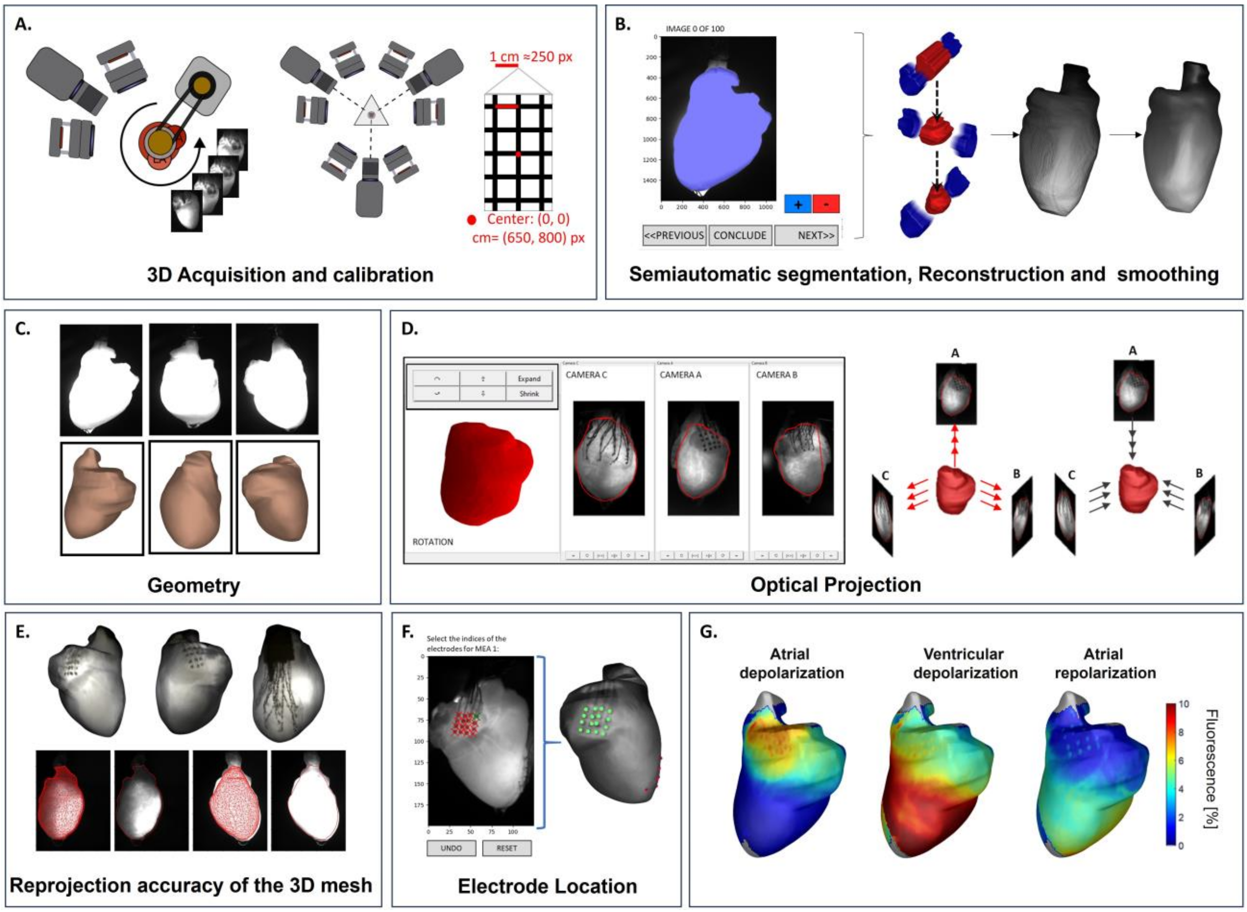

Figure 2

3D acquisition and calibration: A. The optical system acquires images by rotating a single camera 360° around the heart (left). Calibration is performed using a triangular prism placed at the centre of the setup; **B.** Segmentation, reconstruction, and smoothing: A semi-automatic segmentation interface (left) identifies the heart silhouette across all frames. The reconstruction process (centre) carves regions of interest (ROIs) to generate the 3D mesh. Comparison between initial and smoothed meshes (right). **C.** Geometry: refined meshes obtained from the volume boundaries, after applying simplification and filters; **D.** Interface (left) used to align and refine the mapping between optical and geometric data. Inverse projection examples from three cameras are shown (right); **E.** Reprojection accuracy of the 3D mesh: Validation of the alignment between 2D and 3D data using projection contours (bottom row); **F.** Electrode location: python interface to manually identify electrodes location using the 2D optical data (left) and the already computed electrodes over the 3D heart surface (right); **G.** 3D surface projections showing atrial depolarisation (left), ventricular depolarisation (centre), and atrial depolarisation (right).

4. Optical mapping experiments with Blebbistatin can be susceptible to heart swelling, meaning an increase in the overall volume of the heart tissue over the course of the experiment. Have the authors observed any significant increases in the heart's volume or shape over time in their experiments? If so, do these changes influence the spatial mapping between optical and electrical data?

Answer: We appreciate the reviewer’s concern regarding heart swelling due to Blebbistatin administration. While an increase in heart volume was observed, these changes were not accounted for in our results, as similarly done in prior electromechanical optical mapping studies. The final measured heart volume was used for 3D reconstruction and calibration, minimizing any potential impact on spatial mapping. Additionally, the rigid mounting and controlled perfusion helped reduce excessive expansion beyond physiological limits. Given these factors, we consider the influence of swelling on our results negligible. However, future studies could explore dynamic volume tracking to further quantify potential deformations. The following lines were added into the discussion section to solve this concern.

<u>Discussion section (Lines 711-118, pages 25-26)</u>

“Although an increase in heart volume was noted, consistent with previous electromechanical optical mapping studies (Bray *et al.* 2000, Gloschat *et al.* 2018, Kay *et al.* 2004, Lou Ripplinger C. *et al.* 2008, Qu *et al.* 2007), these changes were not accounted for in the current analysis. The final heart volume was used for reconstruction to minimise spatial distortions, and the rigid mounting helped contain expansion within physiological limits. As the focus here is on the development and validation of the experimental platform, such deformations were not corrected, but future work will include enhanced post-processing and dynamic volume tracking to improve spatial accuracy.”

5. Figures 3A and 4A: Are the images of the heart rendered images of the mesh, or are they cropped images directly from the cameras? The sharp and clearly visible electrode wires suggest that these might be original camera images. However, this is not explicitly stated in the text and should be clarified. If the images are indeed rendered from the 3D mesh, please provide

a description of the rendering process. Specifically, are the original camera images texture-mapped onto the mesh triangles, or are the mesh vertices assigned colors?

Answer: Thank you for your observation. We confirm that Figures 3A and 4A are cropped images taken directly from the cameras. These images are not rendered from the 3D mesh, and there is no connection between them and the 3D reconstruction. Regarding image processing, only one image underwent histogram equalization for illustrative purposes, enhancing contrast and improving visibility. This method redistributes pixel intensities to make details more distinguishable, particularly in regions with low contrast. The sharp and clearly visible electrode wires in Figures 3A and 4A reflect the original camera capture. However, we emphasize that all other analyses and data processing were performed using the original intensity matrices without any histogram equalization to ensure consistency and accuracy in quantitative evaluations. To clarify the origin of these images, we have now explicitly stated in the figure captions that they are direct camera images and not generated from the 3D mesh.

6. Figures 3A and 4A: The relationship between the optical traces and the electrode locations needs further clarification. Please specify precisely where the displayed optical traces are extracted from. Is it from the area immediately surrounding a specific electrode? Given that the electrodes are opaque and appear to block optical signals from the underlying tissue, it is unclear why the LAT and phase maps do not show any "blind spots" or missing data corresponding to the electrode locations. Please explain how the optical data is processed to avoid these blind spots. Are the optical traces extracted from the 3D mesh or raw camera images?

Answer: Thank you for your observation. To minimize the influence of noise introduced by the mixed electrode and optical signals, we selected optical traces from regions at least 7 pixels (≈0.5 mm) away from the electrode edges. A binning factor of 8 was applied to the original optical data prior to filtering. This process resulted in a spatial averaging of pixel intensities, leading to the blending of the electrode regions with the surrounding optical signals. While this introduces some inherent noise in the vicinity of the electrodes, it ensures that optical data remains available across the field of view. We clarify that in the preprocessing method section.

<u>Method section - Signal Pre-processing and Filtering (Lines 384-390, page 13)</u>

“A binning factor of 8 was applied to the original optical data prior to filtering. This resulted in spatial averaging of pixel intensities, blending the electrode regions with the surrounding optical signals. Although this introduces some inherent noise near the electrodes, it ensures that optical data remain available across the field of view. Each electrode has a physical size of 1 mm × 1 mm, which, after binning, corresponds to approximately 196 pixels arranged in a 14 × 14 grid. For EGM–optical signal comparison, optical traces were selected from regions located at least 7 pixels (≈ 0.5 mm) away from the electrode edges.”

7. Figures 3B,C and Fig 4B,C: The optical and EGM maps show differences that are difficult to interpret due to their differing visualization geometries. To enable a more meaningful comparison, could the EGM data be mapped onto the reconstructed 3D mesh using the known electrode positions? Furthermore, what factors might contribute to any observed differences in activation patterns or values between the two modalities, even after spatial alignment?

Answer: Following your recommendation, we have mapped the EGM data onto the reconstructed 3D mesh using the known electrode positions. Regarding the LAT maps, we implemented 3D representations (Figure 3B), while also retaining the 2D maps (Figure 4B), in order to illustrate both possible visualisation approaches. The 2D maps were particularly useful for clearly visualising wavefront propagation in the case of electrical mapping. Figures 3C and 4C display the 3D phase maps for both modalities. Potential differences in activation patterns between the two modalities, even after spatial alignment, may arise from differences in signal resolution, tissue-electrode contact, or modality-specific sensitivities to propagation dynamics are presented in the discussion section.

<u>Results section - Panoramic optical and electrical epicardium mapping (Lines 456 - 460, pages 15-16)</u>

> “**Figure 3B**, shows 3D LAT maps for electrical (top) and optical (bottom), for each of the three rhythms. The maps use separate colour schemes for atrial (red) and ventricular (blue) activities, offering insights into the timing and sequence of cardiac electrical activation. Additionally, the phase maps (**Fig. 3C**) provide a dynamic representation of rhythmic patterns, comparing EGM and optical maps side by side.”

<u>Results section – Figure 3 (Lines 491- 504, page 17)</u>

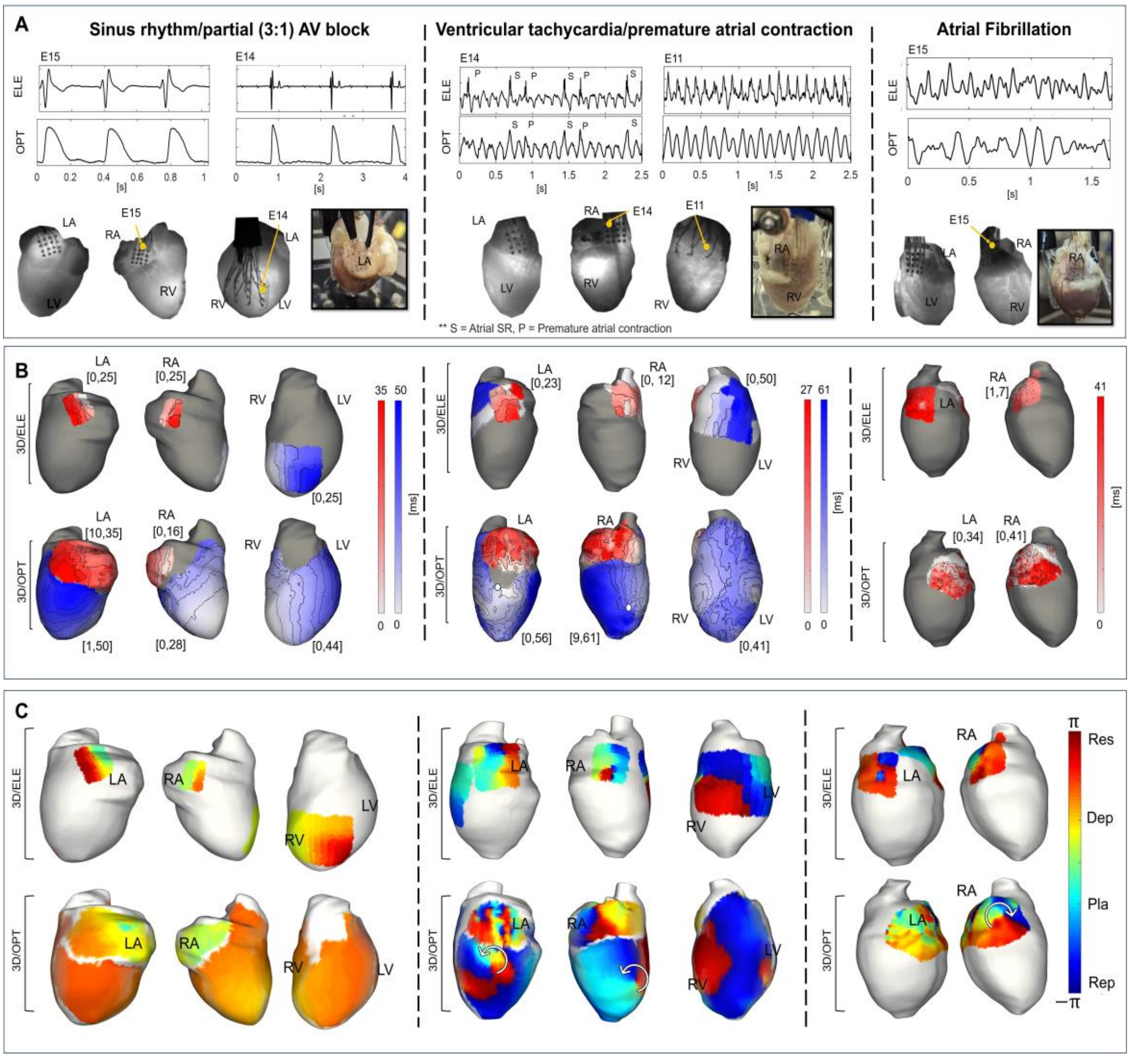

Figure 3

Simultaneous optical and invasive electrical mapping during sinus rhythm with AV block (SR/AVB), Ventricular tachycardia with a premature atrial contraction (VT/PAC), and Atrial Fibrillation with AV block (AF): A. The upper panel shows representative optical and EGM signals recorded from selected regions on the heart surface, illustrating cardiac activity during SR/AVB (left), VT/PAC (middle), and AF (right). In the lower panel, yellow arrows and circles indicate the anatomical locations where these signals were acquired, corresponding to the electrode positions shown in the camera-acquired images; **B.** 3D LAT maps for electrical (top) and optical (bottom) data during SR/AVB (left), VT/PAC (middle), and AF (right). This illustrates the synchronization of data capture and the differentiation between atrial (red) and ventricular (blue) activity. All isochronal lines in LAT are 5 ms apart, except AF with 2 ms; **C.** 3D phase maps for electrical (top) and optical (bottom) data under SR/AVB (left), VT/PAC (middle), and AF (right).

<u>Results section - Panoramic optical and electrical epicardium mapping (Line 517-521, page 18)</u>

"**Figure 4A** (left) shows a stable re-entrant activity over the LV, confirmed also in the respective LAT map, but with a slightly spatial localization of the phase singularity (**Fig. 4B and C**, left). In VF, the HR is 349.5±183.0 bpm in optical recordings and 526.4±227.0 bpm in EGM signals showing

discrepancies between mapping modalities. These LAT maps (**Fig. 4B**) reveal intricate re-entrant patterns across the heart, also observed in phase maps (**Fig. 4C**).”

Results section – Figure 3 (Lines 525- 537, pages 18-19)

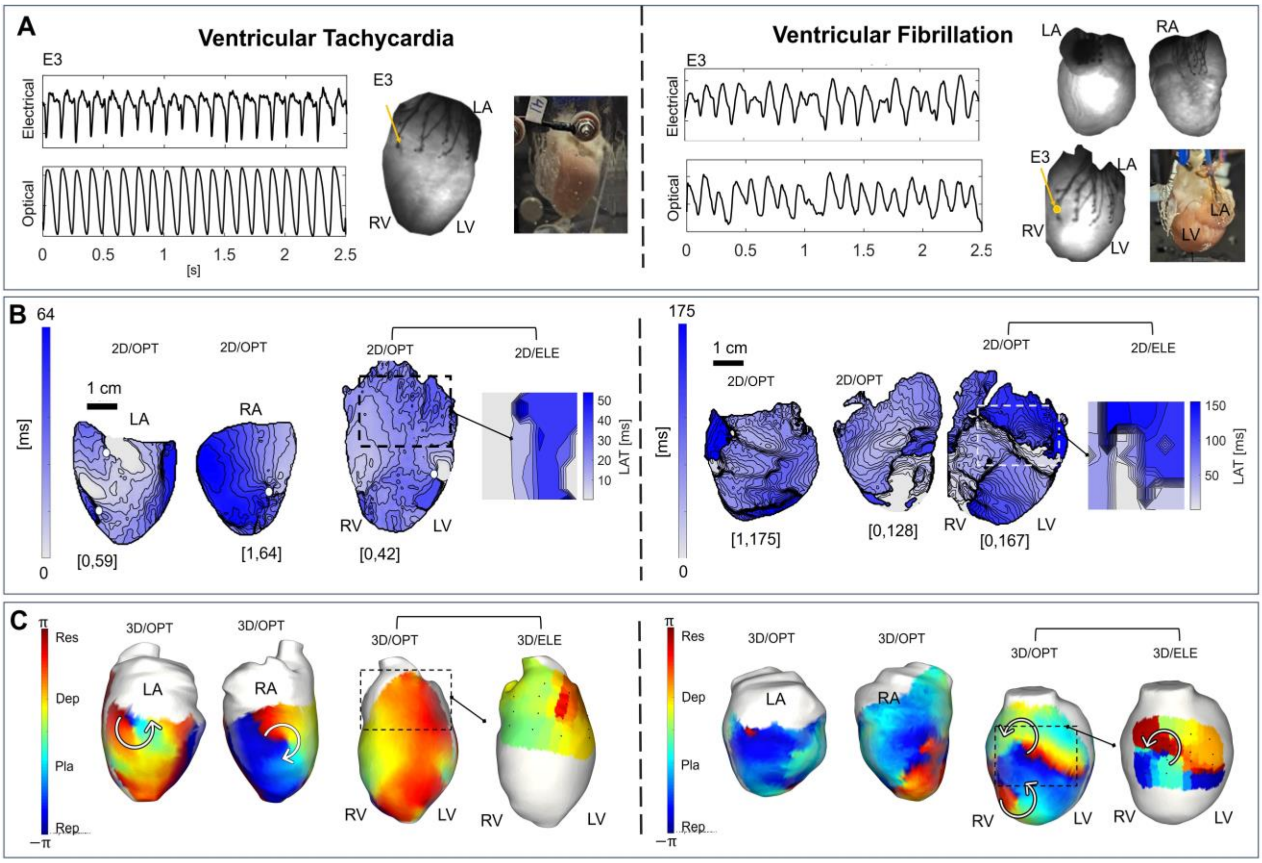

Figure 4

Comparative analysis of VT and VF: A. Electrophysiological recordings and anatomical context. Left: Electrode 3 (E3) voltage signals and fractional change in fluorescence over time during VT, with anatomical position marked on the camera-acquired images. Right: Electrode 3 (E3) voltage signals and fractional change in voltage over time during VF, with electrode position indicated on the camera-acquired images. Locations where the signals were obtained are highlighted with a yellow arrow and circle, corresponding to the electrode positions visualised in multi-angle camera-acquired images; **B.** Optical LAT maps acquired from the three camera views, and contact electrical LAT map from the ventricular MEA, all isochronal lines in LAT are 5 ms apart; **C.** 3D optical phase maps acquired from the three camera views, and a 3D contact electrical phase map from the ventricular MEA.

Discussion section (Lines 703 –711, page 25)

“As preliminary results from the proposed multimodal platform, we present optical and electrical maps in both 2D and as projections onto the reconstructed 3D heart geometry. In the 2D maps, potential differences in activation patterns between modalities (e.g., contact electrical and optical mapping), even after spatial alignment, may arise due to disparities in signal resolution, tissue–

electrode interface quality, or modality-specific sensitivities to propagation dynamics. In the 3D projections, some spatial mismatches were observed, largely attributable to difficulties in distinguishing the microelectrode arrays from the tissue, subtle morphological changes during handling, and structural differences between the perfused and post-perfusion states."

8. An important consideration for the three-camera system is the extent of overlap between their respective fields of view and the resulting uniformity of signal quality across the epicardial surface. Do the three cameras provide sufficient overlap to capture every point on the heart's surface with a good viewing angle for accurate optical mapping? Are there regions, such as the apex or areas on the posterior or lateral walls, where the signal quality might be compromised due to a more oblique or tangential viewing angle from the cameras? In Figure 2H there appear to be areas near the apex where data is missing, and potential artifacts or discontinuities are visible at the transition zones between camera views.

Answer: The three-camera system does not provide complete overlap of the heart's surface, including the apex. To address this limitation, the values where there is no projection were interpolated linearly with the neighbour vertex values, iteratively. To clarify this, the following text was added to the Methods section:

<u>Methods Section -3D heart geometry (Lines 326-331, page 11-12)</u>

"For regions where data from multiple cameras overlap, the overlaid data is combined using a weighted average, where the weight corresponds to the component of each vertex's normal relative to the direction of each camera. For areas with limited overlap, such as the borders and the apex, as well as to smooth transitions between maps, the values where there is no projection were interpolated linearly with the neighbour vertex values, iteratively, to ensure uniformity and accuracy in the resulting optical maps."

9. Supplemental videos demonstrating the temporal evolution of the optical signals (or their phase) on the mesh would significantly enhance the manuscript. This would provide a dynamic visualization of the data and facilitate a more intuitive understanding of the spatiotemporal patterns.

Answer: We appreciate the reviewer's suggestion. We are currently presenting 2D videos of SR and VF camera views as supplementary material. Additionally, the projection of the data onto the 3D mesh over time will be present in another paper under development with focus into discrepancies between optical and electrical mapping for cardiac rhythms characterization.

<u>Results section - Panoramic optical and electrical epicardium mapping (Line 521-523, page 18)</u>

"Videos demonstrating the temporal evolution of the optical signals on the mesh during SR/AVB (SRAVB video) and VF (VF video) are included as Supplemental videos."

10. In section 7.1: "RA presents a slow SR with a CL of 56.44{plus minus}87.82 ms (i.e to 109.22{plus minus}17.22 bpm) and 575.96{plus minus}43.16 ms (i.e to 104.53{plus minus}9.05 bpm) for optical and electrical data, respectively." The first CL value appears to be a misprint, it should be in the range of 600ms. In general, the precision with which the error values are reported throughout the manuscript is often excessive and not justified. I would suggest that the authors revise the number of significant digits used for reporting measured values and their errors to adhere to standard scientific practice.

Answer: Thank you for your observation. We have carefully reviewed all reported values and their associated errors, ensuring that error values are presented with a single significant digit.

<u>Results section - Panoramic optical and electrical epicardium mapping (Lines 473 – 479, page 16)</u>

"The RA exhibits a slow SR with a CL of 556.4 ± 87.8 ms and 575.9 ± 43.1 ms for the optical and electrical data, respectively. The PAC activations (labelled "P" in the plot) occurs following every other atrial sinus activation (labelled "S"), with an interval of 243.2 ± 25.7 ms in the optical signals and 215.9 ± 3.9 ms in the electrical signals, showing a certain difference between the mapping's modalities. Meanwhile, the ventricle displays episodes of VT, with a CL of 123.0 ± 9.3 ms for the optical signals and 127.2 ± 21.7 ms for the EGMs, sustained over a 2.5 s period."

11. In section 7.1 the optical CL values lack sufficient detail regarding the spatial and temporal averaging procedures. Please clarify over what spatial area the optical CL values are averaged. Is it over the entire field of view, specific regions of interest, or individual pixels? Over an entire recording?

Answer: Thank you for highlighting this omission. This clarification has been added to the manuscript.

<u>Methods section - Feature Extraction and Map Generation (Lines 402 – 407, page 14)</u>

"The cycle length (CL) for optical and electrical signals was calculated based on data from 16 specific points, each corresponding to an electrode location. For each electrode, a single pixel was selected from the surrounding neighbourhood of seven pixels closest to the electrode position. For each point, a time window containing ten consecutive activations was chosen, and the mean CL was computed. The final reported CL value represents the average of the measurements from all 16 positions."

12. The panoramic optical mapping data is not shown for the torso tank experiments, where the advantages of 3D mapping, such as spatial information across the entire epicardial surface, versus EGM validation would be most apparent. The optical data should be incorporated into the validation of the rEGM maps, e.g. with a supplementary video comparing the temporal evolution of activation patterns from both optical mapping and ECGI reconstructions.

Answer: We thank the reviewer for the valuable suggestion. We agree that panoramic optical mapping offers important spatial information for validating rEGM maps. In this work, we included comparative analysis of optical signals, tank electrical signals, EGMs and rEGMs in Figures 5 and 6, in the results section.

<u>Results Section-Non-invasive electrical mapping (Torso- Tank) and ECGi (Lines 564– 575, page 20)</u>

> "ECGi analysis is summarized in **Figure 5D** for SR. The left and middle panels of **Figure 5D** display four representative electrodes from different regions from the RA and LA, showing segments 130 ms long, with EGM (black) and rEGM (red) signals overlaid and temporally aligned according to the computed lag. In the RA, electrodes EL2 and EL3 exhibited the highest CC values (0.92), although these were associated with the longest delays (lags around 55 ms). Across all 16 electrodes, lags consistently ranged from 45 to 56 ms, indicating a systematic delay in the reconstructed activation. In contrast, the LA demonstrated better temporal alignment, with lags not exceeding 9 ms. The highest correlation was 0.94 in EL2, with a minimal delay (2 ms), indicating strong morphological and temporal agreement. The boxplot on the right summarises the CC distribution across all electrodes in each region, highlighting slightly greater correlation performance in the LA. An overall mean CC was calculated as the average of 16 signals from the RA and LA, resulting in 0.79 ± 0.09 for the LA and 0.78 ± 0.08 for the RA during SR/AVB in a single beat."

<u>Results Section-Figure 5 (Lines 584 – 596, page 21)</u>

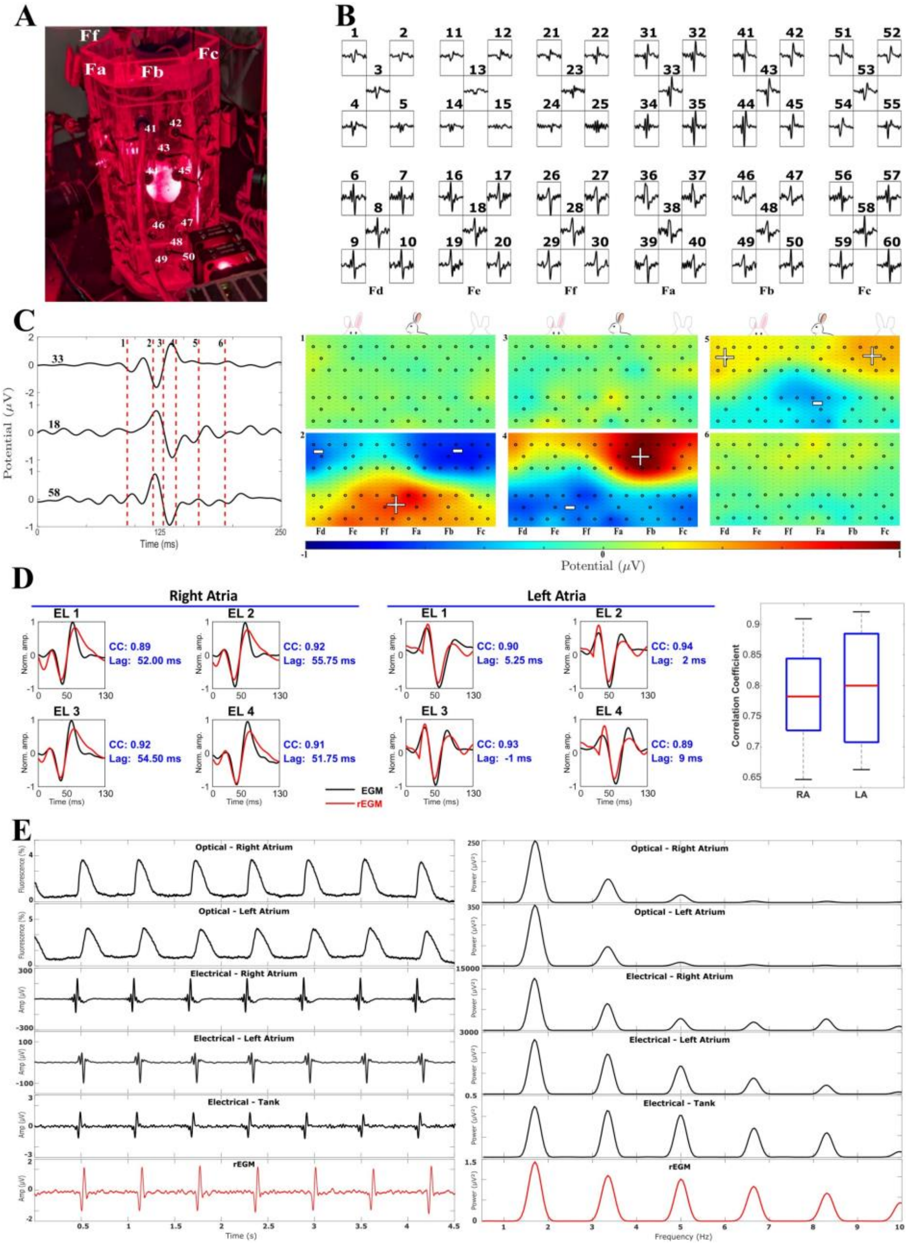

Figure 5

Non-invasive mapping of a SR with AV node blockage. A. Torso-tank setup with labelled faces and electrodes; the heart remains centrally positioned throughout the experiment; **B.** Torso-tank signals during SR, with electrode numbers and tank face labels (Fa–Ff) matching their positions; **C.** 250 ms segment from electrodes 33, 18, and 58 during SR (left); six red lines mark time points for torso-tank potential maps (right), where white ± signals indicate relative potential levels; **D.** CC between EGMs (black) and rEGMs (red). Left and middle: Representative 130 ms segments from four RA and four LA electrodes, with signals overlaid and aligned by computed lag; CC values and lags are shown. Right: Boxplots of CC across all 16 electrodes per region. All signals normalised between –1 and 1; **E.** Left: 4.5 s segment showing, from top to bottom, optical (RA, LA), electrical (RA, LA, tank), and reconstructed (rEGM) signals. Right: Corresponding frequency spectra.

Results Section-Non-invasive electrical mapping (Torso- Tank) and ECGi (Lines 599– 611, page 22)

"A ECGi analysis was also conducted during VT, as shown in **Figure 6**. The 3D potential map of the rEGM (**Fig. 6A**) illustrates a ventricular activation pattern, with the right half of the ventricle displaying more negative potentials, consistent with the wavefront originating from the right side. This spatial pattern agrees with the measured EGM potential map. However, a temporal mismatch is evident in the left region: while electrodes 5–8 are already activated in the rEGM, the EGM signals at electrodes 5 and 6 are still positive, indicating delayed activation in the measured data. Electrodes 1–4 on the left side also show higher magnitude in the EGM rather than in the rEGM. CC analysis was performed for electrodes located on the right side (electrodes 13–16), where the agreement was the highest. The maximum CC reached 0.98, and no temporal lag was observed at these electrodes, indicating very high morphological and temporal similarity. However, as shown in the potential maps, the left side of the ventricle exhibited temporal misalignment. In the rEGM, the activation appears nearly simultaneously across this region, whereas the EGM reflects a more gradual wavefront propagation."

Results Section-Figure 6 (Lines 612-621, page 22)

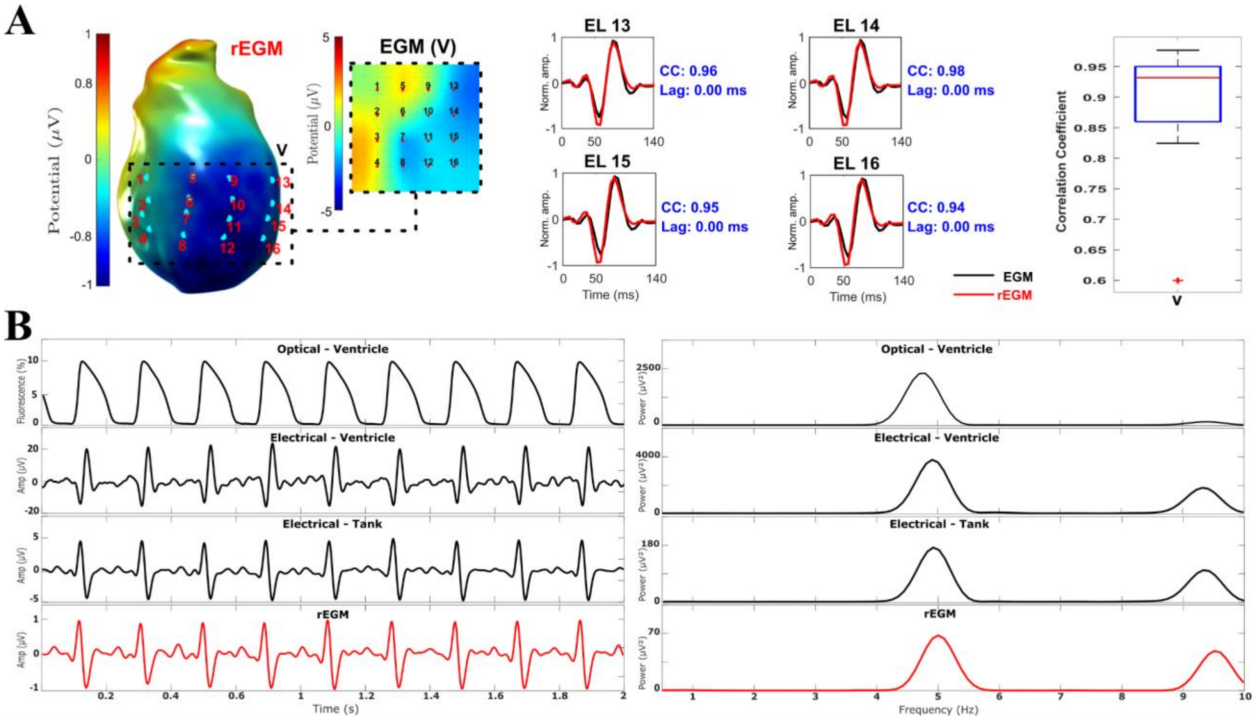

Figure 6

ECGi analysis during VT A. Left: Three-dimensional rEGM potential map with highlighting ventricular electrode positions, alongside the corresponding two-dimensional EGM potential map. Four representative electrodes (EL13–16) are shown with normalised EGM (black) and rEGM (red) signals overlaid, including their CC and lag values. Right: Boxplot of CC values across the 16 ventricular electrodes; **B.** Left: Two-second signal segments recorded from the ventricular region, showing optical fluorescence, measured EGMs, torso-tank signals, and the reconstructed rEGM. Right: Frequency spectra of each signal.

Discussion Section (Line 719-730, page 26)

"In our study, the comparison between rEGMs and EGMs showed generally good agreement (typically above 0.7). A good match (defined as CC > 0.70) was observed in 78% of RA electrodes and 76% of LA electrodes. These values are comparable to previous findings in the literature. Cluitmans *et al.* (2017) reported a mean CC of 0.71 in anaesthetised dogs using body-surface and epicardial recordings, while Ghosh *et al.* (2005) demonstrated a mean CC of 0.81 in torso-tank experiments with explanted canine hearts over multiple beats. Under SR/AVB conditions, analysis of the LA revealed that some neighbouring electrodes exhibited distinct signal morphologies despite their spatial proximity. This local variability contributed to lower CC values (below 0.7) in certain electrodes, likely due to limitations of the regularisation method, which struggles to capture abrupt spatial variations. Moreover, suboptimal contact between the MEA and the epicardial surface may have introduced non-physiological distortions in the recorded signals, contributing to both reduced CC values and the lags consistently observed in the RA."

13. The reconstructed electrogram maps in Figure 5D exhibits a degree of "graininess" or "roughness" that deviates from the smooth spatial gradients typically expected in electrophysiological signals. Could you comment on the factors that contribute to this? This seems to contribute the lag of the rEGM of electrode 16. The EGM/rEGM plots should also be plotted on top of each other so that they can be compared more directly.

Thank you for your observation. The accuracy of ECGI reconstructions is indeed highly sensitive to noise in the measured signals due to the ill-posed nature of the inverse problem. The observed "graininess" or "roughness" in the reconstructed electrogram maps can be attributed to several factors. Firstly, the use of zero-order Tikhonov regularization prioritizes minimizing the magnitude of the solution but does not enforce spatial smoothness between neighbouring nodes. As a result, this approach can lead to abrupt or spatially disjoint variations in the reconstructed maps. Additionally, suboptimal selection of the regularization parameter (λ) may further compromise the smoothness and accuracy of the solution. Another contributing factor is noise present in the torso tank signals, which is amplified during the interpolation and inverse reconstruction process. Furthermore, anatomical factors such as electrode placement on the heart's curvature may lead to variable tissue contact, signal distortion, and temporal misalignment. We have taken steps to mitigate these effects by improving the preprocessing of the tank signals prior to the ECGi reconstruction and by enhancing the visual presentation of the reconstructed maps through surface smoothing techniques to reduce abrupt transitions in the display. Specifically, we downsampled the signals from 4000 Hz to 200 Hz, performed the reconstruction at this lower resolution, and subsequently up sampled the results back to 4000 Hz for direct comparison with the epicardial mapping. This approach not only led to a smoother and more physiologically consistent visualization of the reconstructed signals but also reduced the computational resources required for the reconstruction process. Finally, in response to the reviewer's suggestion, we have updated Figure 5D and Figure 6A to display the EGM and rEGM signals plotted on top of each other. This adjustment facilitates a more direct and meaningful comparison of the signal morphology between measured and reconstructed data. The changes in preprocessing are now described in the Methodology Section-Electrocardiographic Imaging, and the

discussion of the observed graininess in the reconstructed maps has been addressed in the Discussion section.

Discussion section (Lines 730-736, page 26)

"The "graininess" or spatial roughness observed in some rEGM maps may also reflect these limitations. This effect may arise from the use of zero- order Tikhonov regularisation, which minimises signal magnitude without promoting spatial smoothness. In addition, noise in the torso-tank signals and anatomical variability may contribute to abrupt variations in the rEGM maps. Frequency analysis further demonstrated that the reconstruction preserved the frequency components of the signals when compared with the optical, EGMs, and tank signals in both sinus AVB and sinus tachycardia recordings."

14. The manuscript focuses on sinus rhythm to evaluate the rEGM signals, which has a very fast and spatially uniform activation pattern and does not reflect the complexity of clinically relevant arrhythmias. Validating ECGI during pacing or tachycardia, which are more representative of clinically important scenarios, would greatly enhance the impact of this manuscript.

Answer: Thank you for this important comment. We agree that evaluating ECGI in more complex and clinically relevant scenarios is essential to demonstrate its robustness and broader applicability. To address this, we have added a new figure, Figure 6 (previously presented in question 12 by the reviewer) that presents an analysis of the ventricular tachycardia, which represents a more common and clinically relevant rhythm compared to baseline sinus rhythm. This figure includes the optical signals, MEA recordings, tank signals, and the 3D rEGM map. We have also included representative electrode signals with their corresponding cross-correlation coefficients, providing a quantitative comparison of signal morphology across modalities. In addition, we are actively working on the reconstruction and validation of ECGi during different types of arrhythmias, including more complex pacing protocols and tachyarrhythmias.

15. The manuscript would benefit from a more explicit and detailed discussion of the advantages of panoramic optical mapping. Furthermore, a more thorough review of previous work in this area is needed, particularly regarding how the authors' approach differs from and potentially improves upon earlier methods. For example, the seminal studies by Bray et al. (2000) DOI: 10.1109/10.871412 or Kay et al. (2004) DOI: 10.1109/TBME.2004.827261 appear to be pertinent to this approach and should be mentioned or discussed.

To address this comment, the following text has been added to the manuscript:

Discussion section (Lines 659 – 678, page 24)

"One of the earliest implementations was developed by Bray *et al.* (2000), who utilised two planar, front-surface mirrors to achieve panoramic optical mapping in rabbit hearts using a single CCD

camera operating at 335 frames per second. Although this three-view approach was cost-effective, it considerably reduced spatial resolution, yielding only 128 × 64 pixels per projection of the heart. Subsequently, Kay *et al.* (2004) extended this methodology to larger mammalian hearts, specifically porcine models, employing a single mirror and two CCD cameras to acquire four views at a frame rate of 250 frames per second. Later, Qu *et al.* (2007) proposed the use of photodiode arrays (PDA) instead of CCD cameras, enhancing the system's temporal resolution by increasing the sampling frequency to 5000 frames per second in a three-view system, with a spatial resolution of 769 pixels (using three 16 × 16 PDAs). Both Kay *et al.* (2004) and Qu *et al.* (2007) utilised higher spatial resolution cameras compared to Bray *et al.* (2000) for three-dimensional (3D) heart reconstruction, requiring a more comprehensive calibration process. Finally, Lee *et al.* (2017) developed a low-cost panoramic optical mapping system using USB 3.0 CMOS cameras, aiming to make optical mapping more accessible to research laboratories. The system implemented in this study operates three optical cameras at a sampling frequency of 500 Hz, which is sufficient for the acquisition of cardiac arrhythmias, with an original spatial resolution of 1600 × 1000 pixels. Depending on the specific study requirements, this resolution can be reduced using binning to facilitate processing or retained in its original form for specialised analyses. Additionally, the use of the same camera for both fluorescence imaging and 3D reconstruction eliminates the need for an extended calibration process, thereby streamlining data acquisition and analysis. "

Minor points:

1. The manuscript states that the 3D geometry is saved in five resolutions (20000, 10000, 5000, 2500, and 1200 vertices). What is the purpose of using different mesh resolutions? The manuscript does not clarify which resolution is used in the subsequent analysis.

Answer: Thank you for your comment. The 3D heart geometry resolution is 20,000 vertices. This was corrected in the text.

<u>Discussion section (Lines 307-311, page 11)</u>

"**Figure 2C** shows three refined meshes, corresponding to each camera, obtained from the volume boundaries, after applying simplification and filters. Assuming a reconstruction with 20,000 vertices covering an average area of approximately 2902 ± 400 mm², the average resolution per vertex is around 0.15 ± 0.2 mm², which corresponds to a linear spacing of approximately 0.54 ± 0.07 mm between vertices."

2. How strongly is the mesh smoothed during the refinement? Could you perhaps show the difference between the raw and the refined mesh?

Answer: A simple smoothing was applied with 5 iterations. The comparison between the raw and refined meshes has been added as supplementary material and cited in the manuscript as follows.

<u>Methods section - 3D heart geometry (Lines 285 – 289, page 10)</u>

“**Figure 2**B (right) illustrates the improvement in mesh quality achieved through surface refinement (5 iterations) of a 3D reconstructed heart model, displaying the raw mesh obtained from initial segmentation and the same model after applying a smoothing algorithm, resulting in a more anatomically realistic and visually coherent representation.”

3. What is the bit depth of the camera images? 12-bit?

Answer: Yes, the camera images have a bit depth of 12 bits. This information has been added to the manuscript for clarification.

Methods section - Setup description (Lines 246-248, page 9)

“Recordings were acquired simultaneously at 500 frames per second, with a resolution of 1600 × 1000 pixels, 12-bit depth, and a total duration of 10 seconds (yielding 5000 frames), using Norpix-StreamPix 9 software.”

4. LAT abbreviation is used in 6.7 before it has been introduced.

Answer: The abbreviation "LAT" is now properly introduced in the local activation time section in the manuscript.

5. The description of the ECGI reconstruction process could benefit from further detail to ensure the methodology is clear and reproducible.

Answer: Thank you for your suggestion. To provide greater clarity and ensure the methodology is clear and reproducible, we have added the following lines to the

Methods section - Electrocardiographic Imaging (Lines 349-372, pages 12-13)

“ECGi enables the reconstruction of epicardial electrograms (rEGMs) from non-invasive measurements. In this approach, epicardial potentials are estimated by solving the Laplace equation within the volume conductor formed by the torso-tank, based on the measured torso potentials and the geometric correspondence between the 3D surfaces of the heart and torso (Salinet *et al.* 2021). Preprocessing of the tank signals starts with Laplacian interpolation to enhance spatial resolution from 60 to 218 electrodes, ensuring better alignment with the tank geometry (Oostendorp *et al.* 1989). Baseline drift is corrected using an adaptive cubic spline filter (Sörnmo *et al.* 2005), followed by a sixth-order Butterworth low-pass filter with a cut-off frequency of 30 Hz to attenuate high-frequency noise. This cut-off was chosen based on spectral analysis using the Fast Fourier Transform (FFT). To improve computational efficiency without compromising signal integrity, the signals are downsampled from 4000 Hz to 200 Hz. Subsequently, Principal Component Analysis (PCA) is applied to extract the most relevant components (Semmlow *et al.* 2014), providing a compact and meaningful representation for use in ECGi reconstruction.

ECGi pipeline involved discretising the heart and torso surfaces into triangular meshes, allowing for a linear model to define the relationship between potentials on both surfaces. This relationship is expressed through the transfer matrix (Salinet *et al.* 2021; Rudy 2013), computed using the Boundary Element Method (BEM), which incorporates both the geometric structure and electrophysiological characteristics of the volume conductor (Salinet *et al.* 2021). The inverse problem in electrocardiography is inherently ill-posed, meaning that small errors in torso potential measurements or geometry can lead to large inaccuracies in the reconstructed epicardial potentials (Rudy 2013; Cluitmans *et al.* 2015). To address this instability, the inverse solution was obtained using zero-order Tikhonov regularisation, currently regarded as the gold standard in ECGi (Tikhonov *et al.* 1977). The optimal regularisation parameter (λ) was selected using the L-curve method, which balances fidelity and smoothness to minimise reconstruction error (Hansen *et al.* 1993)."

Methods section - Feature Extraction and Map Generation (Lines 437- 442, page 15)

"Cross-correlation (CC) is used to assess signal morphology similarity between EGMs and rEGMs, as it quantifies shape agreement independently of amplitude differences (Cluitmans *et al.* 2018). To ensure consistency, both signals were normalised prior to analysis (Wang *et al.* 2021). A moving mean filter was applied to the rEGM signals to reduce artefacts and enhance activation clarity. Electrode positions were localised using optical mapping, and the corresponding nodes on the 3D heart geometry were identified, enabling direct spatial comparison of EGM and rEGM signals."

6. Many journal names in the references are not correctly capitalized.

Answer: Thank you for your observation. The capitalization of journal names in the references has been reviewed and corrected in the manuscript.

REFEREE COMMENTS

Referee #2:

The manuscript from Siles et al. describes a new system for simulataneously performing optical mapping, ECGi and multi-electrode mapping on a Langendorff-perfused rabbit heart. The approach is interesting and the description likely to be of benefit to the community. The manuscript would benefit from some additional analysis and further copy editing, but I have no major concerns.

1. The torso tank seems relatively small relative to the size of the heart. How does this compare to the relative size of heart and torso for humans and what effect will this have?

Answer: The torso tank may appear relatively small in comparison to the heart; however, it is designed to replicate the rabbit's thorax and part of the abdomen, as detailed below. It is important to note that the current study focuses exclusively on replicating the geometry and volume of the rabbit torso; therefore, no direct comparison to human thoracic dimensions or scaling effects is intended at this stage. To address this point, a supporting information figure (<u>review purposes only and not for final publication</u>) has been included to illustrate the rationale behind the tank design and its anatomical relevance to the rabbit model.

<u>Supporting information - Comparison between the rabbit torso and the experimental tank volumes (Lines 1080-1091, page 34)</u>

"The developed setup was designed for New Zealand White rabbits with an average body weight between 3 and 4 kg. To date, a total of 18 rabbit specimens have been considered, with an average body weight of 3.4 ± 0.3 kg, approximated to ∼3 kg. The average heart weight was 9.9 ± 1.9 g, rounded to ∼10 g. Figure 7A presents a representative rabbit alongside a cylindrical model with a circumference of 32 cm and a height of 18 cm, used to estimate the total body volume. This calculation resulted in an estimated volume of 1466.77 cm, approximated to ∼1500 cm. The experimental tank, designed as a hexagonal prism with a side length of 6 cm and a height of 18 cm, has a total volume of 1683.55 cm, approximated to ∼1700 cm **(Fig. 2B).** The representative heart shown in **Figure 2C** exhibits a volume of 10 ml, assuming an average tissue density of approximately 1.0 kg/L. The heart-to-torso volume ratio in rabbits was calculated as 10 ml / 1500 ml, yielding 0.67%. Similarly, the heart-to-tank ratio was determined as 10 ml / 1700 ml, yielding 0.59%. The similar volumes of the torso and the tank support the use of the tank as a physiological surrogate during experiments."

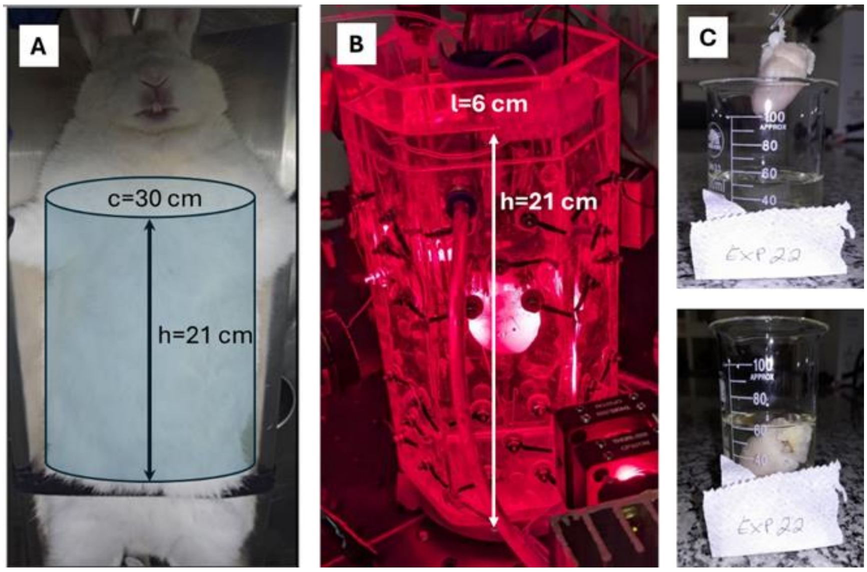

Figure 2 (not included in the paper)

Comparison between the rabbit torso and the experimental tank volumes. A. A representative New Zealand White rabbit showing the cylindrical approximation used for torso volume estimation (circumference = 32 cm, height = 18 cm). **B.** Hexagonal experimental tank (side length = 6 cm, height = 18 cm) designed to match the estimated torso volume. **C.** Excised rabbit heart used for volume measurements.

2. The extent of interpolation for both the MEA and surface tank is extremely large (nearly 14x for MEA and 20x for the surface tank). Is this valid? Can you compare with optical mapping or a recording done with more electrodes (even if not done simultaneously)?

Answer: We acknowledge the reviewer's observation regarding the extent of interpolation applied to both the MEA and the surface tank data. As the signal processing pipeline for both datasets is still under development, the current interpolation factors (approximately 14× for the MEA and 20× for the tank surface) were selected to enable an initial visual comparison with optical mapping data. We recognise that this approach requires further refinement, and a more rigorous validation—ideally involving high-density recordings or simultaneous optical-electrical comparisons—is planned for future studies. Moreover, the level of interpolation done in this study is not so different than done by the commercial systems, where a massive interpolation is required due to the restricted number of electrodes to the catheters, where the maximum is 64 electrodes to cover atrial or ventricle chambers of humans. For non-invasive mapping, body surface potential mapping studies in literature has shown that above 32 electrodes are sufficient to cover complex rhythms such atrial fibrillation. Several research groups use 64-based systems. Commercial systems for ECGi use between 128 to 256 for humans. We are using a torso-tank model with 60 electrodes to a rabbit model, more than required, according to the expertise of this research group.

3. The correlation of results seems very good between systems. It would be good to also examine a spatial correlation especially between ECGi and optical mapping. Perhaps something such as the similarity or nearness of phase singularity positions would be beneficial. It seems ECGi

would smooth this and likely position further away, but that would perhaps highlight the advantage of this system in that you could quantify under what conditions ECGi or MEA can accurately track phase singularities.

Answer: Thank you for your suggestion. The focus of this study was to present the multi-model mapping platform for cardiac studies. Once we validated, we are working in a broader study to highlight discrepancies between mapping modalities across regular and complex rhythms.

Minor comments:

1. The results section is a bit too descriptive, describing exactly what is happening in the figures rather than summarizing them.

Answer: We thank the reviewer for this valuable observation. We revised the text to provide more concise summaries of the figures.

2. Fig. 5 - the numbering of electrode 16 does not fit with the general numbering and makes the example slightly confusing. Perhaps the numbering could be altered to be standardized or a different example face could be shown as all others number sequentially. Also in panel C showing a schematic of the heart rather than of a rabbit may help the readers to better orient themselves.

Answer: Thank you for your observation. To improve the presentation of the results, the numbering of the electrodes has been reorganized to follow a standardized sequential order, both in the figure and in the text. This adjustment ensures greater clarity and consistency across the manuscript.

3. In the discussion it is highlighted that optical mapping cannot capture electrical potentials the same way as clinical tools do. I think this should be rephrased slightly as it implies this is a disadvantage, whereas it's more just a symbol of the differences in the systems and optical mapping is better in that you see true transmembrane potential dynamics. I would instead just highlight that direct comparisons with clinical measurements is challenging as relating transmembrane potentials to extracellular potentials isn't necessarily straightforward.

Answer: We thank the reviewer for this insightful suggestion. We agree that the original phrasing could imply a disadvantage and have revised the text to better reflect the complementary nature of optical and clinical recordings. The following paragraph has been added to the Discussion section.

<u>Discussion section (Lines 683-687, pages 24-25)</u>

“While optical mapping provides high- resolution visualisation of cellular-level activity and action potential propagation, it measures transmembrane potentials, which are fundamentally different from the extracellular potentials captured by clinical tools such as ECG or body surface potential mapping

(BSPM). This intrinsic difference makes direct comparisons challenging but also highlights the complementary nature of these modalities.”

REFEREE COMMENTS

Referee #3:

Paredes et al. provide the first description of a novel system developed by their group that allows the simultaneous acquisition of optical mapping, electrogram and ECGi based mapping in in vivo Langendorff style preparations. The paper is well-written, and substantially adds to the field by developing novel methods that are a major advance over existing tools. The methods are described well overall, and the techniques are a sufficient advance in my view to warrant a specific manuscript to describe them. My only concern is whether there is sufficient detail to allow replication experiments. In particular, I think more detail or planning diagrams would be helpful to allow the tank setup to be replicated. The paper of Lee et al 2017 cited by the authors is an example manuscript in the field that comparably aimed to allow other investigators to replicate the scientific experiments. I think more detail will ultimately benefit the authors by allowing additional investigators to map atrial and ventricular arrhythmias as described by the authorship group.

Answer: Thank you for your feedback about our work. Following your suggestion, we have updated the manuscript to include a new schematic image providing more detail of the tank setup. We believe this additional figure will help clarify the system’s configuration and facilitate replication by other groups.

Method section - Setup description (Lines 174 – 249, pages 6-9)

“This study describes an experimental platform for mapping the cardiac electrical activity in Langendorff-perfused New Zealand White rabbit hearts. The system combines synchronized high-speed optical mapping with simultaneous electrical mapping to characterise cardiac arrhythmias (**Figure 1A**). Electrical mapping includes a torso-tank model for non-invasive mapping, with electrodes placed on the tank containing the Langendorff-perfused heart (torso-tank rabbit model), and epicardial electrical mapping using customised multi-electrode arrays in contact with both atria and ventricles. The following section provides a more detailed description of the implemented setup. **Figure 1B** shows the top-view photograph of the experimental setup mounted on the optical table, highlighting the positioning of cameras.

1.1.1. Epicardial electrical mapping. Three custom-designed multi-electrode arrays (MEAs) with 16 electrodes were developed, and placed in the LA (#1), RA (#2), and ventricle surface (#3) for epicardial electrical mapping (**Fig. 1A, light purple #1,2 and 3**). For the atria, the 16 platinum iridium electrodes sourced from a commercial catheter were fixed onto a 4 × 4 configuration and fixed in a polyethylene terephthalate (PET) square of 14 × 9.5 mm with an inter-electrode distance of 2 mm. The ventricular MEA used the same number of electrodes, but they were arranged on a larger PET square, measuring 19 × 22 mm with an inter-electrode distance of 3 mm vertically and 6

mm horizontally. Each MEA was grounded via a metal ring fixed between the Langendorff cannula and the heart's aorta. The MEAs were connected to a 64-channel recording head-stage (RHD 64 Channel Recording Head-stages, C3315, Intan Technologies) via 36-pin wire adapters (C3420, Intan Technologies). These connections linked MEAs to Open Ephys acquisition board, enabling the acquisition of the epicardial EGMs at a sampling rate of 4 kHz.

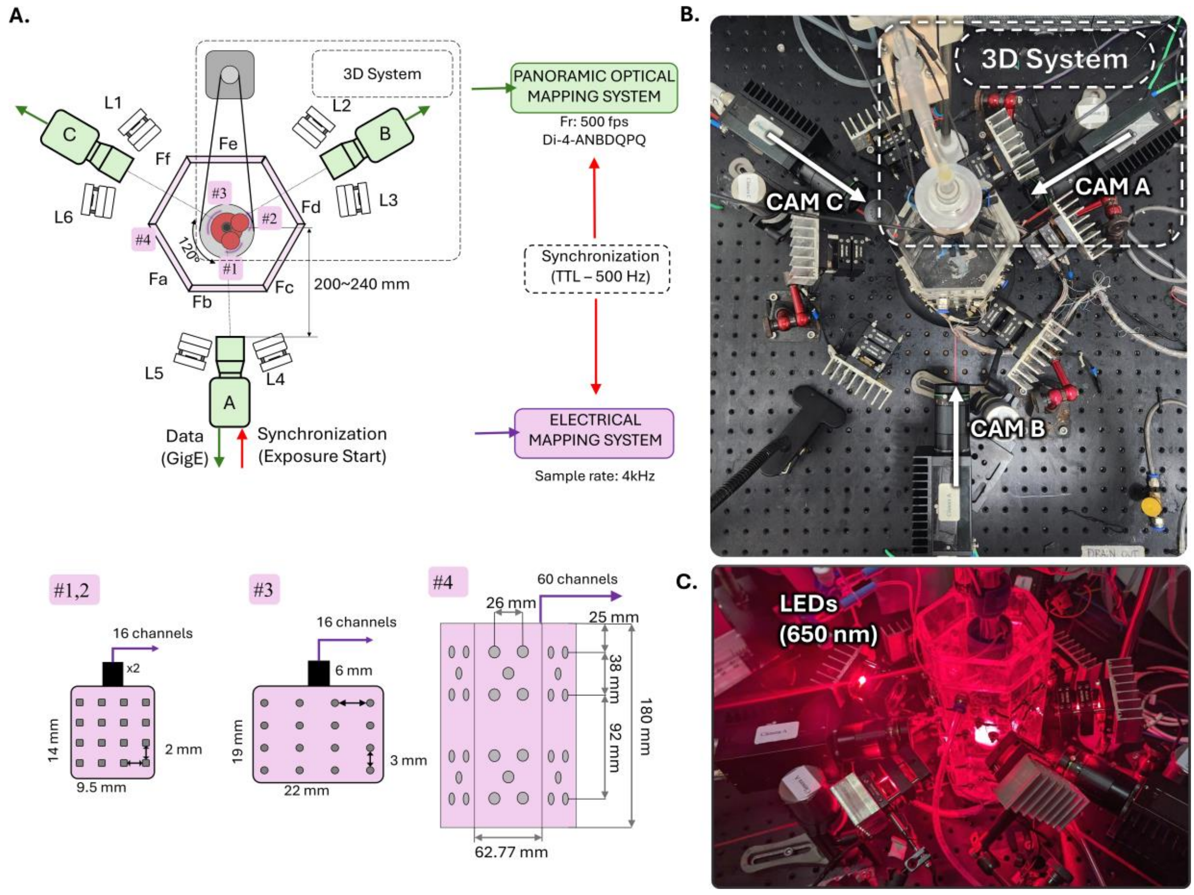

Figure 1

Schematic diagram and photographs of the integrated experimental setup for simultaneous panoramic optical and electrical mapping. A. Schematic representation of the three-dimensional (3D) system integrating the optical mapping system (light green) and the electrical mapping system (light purple). Three high-speed cameras (Cam A, B, and C) are positioned around the Langendorff-perfused heart for panoramic optical mapping. The electrical mapping system includes three custom-designed multi-electrode arrays (MEAs), labelled #1 to #3; and a torso-tank model for non-invasive mapping labelled #4. These MEAs are placed over the RA, LA, and ventricular surface, respectively. Detailed representations of the MEAs, including their real dimensions, are shown at the bottom of the schematic; **B.** Top-view photograph of the experimental setup mounted on the optical table, highlighting the positioning of cameras. A dashed box indicates the custom-built 3D support structure designed to acquire the sequence of images for the 3D reconstruction with the optical camera A; **C.** Photograph of the system during an optical mapping experiment, showing red LEDs (650 nm) illuminating the heart.

1.1.2. Torso-tank model for non-invasive electrical mapping. The torso-tank model is designed to simulate the physical properties of the rabbit's torso. This calculation resulted in an estimated volume of approximately 1500 cm for the rabbit's torso and the designed tank. The model is a hexagonal tank (side length = 62.77 mm, height = 180 mm) designed for high-density, non-invasive electrical mapping (Fuller *et al.* 2000). Ten unipolar surface-mounted electrodes were strategically placed on each face of the hexagonal tank (**Fig. 1A #4**). These electrodes, made of 10 mm diameter stainless steel bolts, were positioned to create clear view windows ensuring unobstructed views of the heart for panoramic optical mapping, as described in the next section. The reference electrode for each surface-mounted electrode was connected via a soldered metal strip attached to the Langendorff cannula in the aorta. A total of 60 electrodes were connected to a 64-channel recording head stage (RHD 64 Channel Recording Head stages, C3315, Intan Technologies) through 36-pin wire adapters (C3420, Intan Technologies). This configuration enabled high-density electrocardiogram acquisition at a 4 kHz sampling rate, using an Open Ephys acquisition board and the open-source Electrophysiology Software. The heart was immersed in the tank and filled with a high concentration sucrose solution (23 mM NaCl, 260 mM sucrose, pH 6.8±0.4, and conductivity of 2.23 μS/cm). This solution mimics the conductivity of the animal's chest cavity and was continuously recirculated using a temperature-controlled ($37 - 38$ °C) water bath.

1.1.3. Panoramic optical mapping. Six deep red (650 nm) LEDs (Luminus Devices) provided uniform illumination of the curved heart surface (**Fig. 1C**). The LED light was collimated using a condenser lens (ACL2520U-A, Thorlabs) and passed through a band-pass filter (650/40 nm, Thorlabs). Fluorescence emitted from the heart was filtered using a 715 nm long-pass filter (FF01-715/LP-22-D, Semrock, USA) before being captured by three high-speed cameras (HB-1800-S, Emergent Technologies) equipped with C-mount 25 mm lenses (Fujinon CF25ZA). The intrinsic parameters also include a pixel size of 9 × 9 μm and a sensor size of 1.1" (17.6 mm CMOS). The lens has a focal length of 25 mm and a distortion smaller than 1% within the region of interest (ROI), which was considered negligible. Cameras were placed at a height of 9 cm above the base, positioned 120° apart around the tank. Their radial distance (R) from the tank centre varies between 20 cm and 24 cm, according to the heart's size, forming an annular arrangement instead of a fixed circular configuration. The coordinates for each camera in the world reference frame are expressed as follows:

$$Camera\ A\!:\ (R, 9, 0)\,,\text{with } 20\ \leq\ R\ \leq\ 24\text{ cm} \tag{1}$$

$$Camera\ B\!:\ \left(-\frac{R_2}{2}, 9, \frac{\sqrt{3}R_2}{2}\right),\text{with } 20\ \leq\ R\ \leq\ 24\text{ cm} \tag{2}$$

$$Camera\ C\!:\ \left(-\frac{R_2}{2}, 9, -\frac{\sqrt{3}R_2}{2}\right),\text{with } 20\ \leq\ R\ \leq\ 24\text{ cm} \tag{3}$$

All three cameras visualised the ventricles, with additional coverage of the LA by Camera A and the RA by Camera B. Recordings were acquired simultaneously at 500 frames per second, with a resolution of 1600 × 1000 pixels, 12-bit depth, and a total duration of 10 seconds (yielding 5000 frames), using Norpix-StreamPix 9 software. The cameras were triggered via hardware using the GPI5 input and automatically stopped after 10 seconds (**Fig. 1A**, light green)."

Dear Dr Salinet,

Re: JP-TFP-2025-287815R1 "An Integrated Platform for 2D and 3D Optical and Electrical Mapping of Arrhythmias in Langendorff-Perfused Rabbit Hearts" by Jimena Gabriela Siles Paredes, Vinícius Silva, Tainan Neves, Italo Sandoval, Angélica Drielly Santos de Quadros, Giovanni Weber, Óscar Barquero, Ilija Uzelac, and João Salinet

We are pleased to tell you that your paper has been accepted for publication in The Journal of Physiology.

TRANSPARENT PEER REVIEW POLICY: To improve the transparency of its peer review process The Journal of Physiology publishes online, as supporting information, the peer review history of all articles accepted for publication. Readers will have access to decision letters, including Editors' comments and referee reports, for each version of the manuscript, as well as any author responses to peer review comments. Referees can decide whether or not they wish to be named on the peer review history document.

The last Word (or similar) version of the manuscript provided will be used by the Production Editor to prepare your proof. When this is ready you will receive an email containing a link to Wiley's Online Proofing System. The proof should be thoroughly checked and corrected as promptly as possible.

Authors should note that it is too late at this point to offer corrections prior to proofing. Major corrections at proof stage, such as changes to figures, will be referred to the Editors for approval before they can be incorporated. Only minor changes, such as to style and consistency, should be made at proof stage. Changes that need to be made after proof stage will usually require a formal correction notice.

All queries at proof stage should be sent to: TJP@wiley.com

Are you on Twitter? Once your paper is online, why not share your achievement with your followers? Please tag The Journal (@jphysiol) in any tweets and we will share your accepted paper with our 30,000 followers!

If you would like to receive our 'Research Roundup', a monthly newsletter highlighting the cutting-edge research published in The Physiological Society's family of journals (The Journal of Physiology, Experimental Physiology and Physiological Reports), please click this link, fill in your name and email address and select 'Research Roundup': https://www.physoc.org/journals-and-media/membernews/

Yours sincerely,

Bjorn Knollmann
Senior Editor
The Journal of Physiology

P.S. - You can help your research get the attention it deserves! Check out Wiley's free Promotion Guide for best-practice recommendations for promoting your work at www.wileyauthors.com/eeo/guide. You can learn more about Wiley Editing Services which offers professional video, design, and writing services to create shareable video abstracts, infographics, conference posters, lay summaries, and research news stories for your research at www.wileyauthors.com/eeo/promotion.

IMPORTANT NOTICE ABOUT OPEN ACCESS: To assist authors whose funding agencies mandate public access to published research findings sooner than 12 months after publication The Journal of Physiology allows authors to pay an Open Access (OA) fee to have their papers made freely available immediately on publication.

The Corresponding Author will receive an email from Wiley with details on how to register or log-in to Wiley Authors Services where you will be able to place an order.

You can check if your funder or institution has a Wiley Open Access Account here: https://authorservices.wiley.com/author-resources/Journal-Authors/licensing-and-open-access/open-access/author-compliance-tool.html

----
EDITOR COMMENTS

Reviewing Editor:

Your manuscript was assessed again and all expert referees confirmed that their concerns have been addressed sufficiently.

Senior Editor:

The revised manuscript is acceptable for publication. Thank you for your excellent contribution to the Journal!

REFEREE COMMENTS:

Referee #1:

I thank the authors for their thoughtful and comprehensive response. The manuscript has been significantly improved by the additional analysis, revised figures, textual revisions, and the inclusion of supplementary videos. All key points raised in the review have been addressed satisfactorily. In my opinion, the paper is now ready and well-suited for publication in The Journal of Physiology.

Referee #2:

Authors have addressed all my concerns.

Referee #3:

My concerns with the paper are substantively addressed by the revisions made by the authors.
